# Metatranscriptomic and functional metagenomic analysis of methylphosphonate utilization by marine bacteria

**DOI:** 10.3389/fmicb.2013.00340

**Published:** 2013-11-26

**Authors:** Asunción Martínez, Laure-Anne Ventouras, Samuel T. Wilson, David M. Karl, Edward F. DeLong

**Affiliations:** ^1^Division of Biological Engineering, Department of Civil and Environmental Engineering, Massachusetts Institute of TechnologyCambridge, MA, USA; ^2^Center for Microbial Oceanography: Research and Education (C-MORE), University of HawaiiHonolulu, HI, USA; ^3^Department of Oceanography, School of Ocean and Earth Science and Technology, University of HawaiiHonolulu, HI, USA

**Keywords:** methylphosphonate, phosphonates, genomics, transcriptomics, functional screens

## Abstract

Aerobic degradation of methylphosphonate (MPn) by marine bacterioplankton has been hypothesized to contribute significantly to the ocean's methane supersaturation, yet little is known about MPn utilization by marine microbes. To identify the microbial taxa and metabolic functions associated with MPn-driven methane production we performed parallel metagenomic, metatranscriptomic, and functional screening of microcosm perturbation experiments using surface water collected in the North Pacific Subtropical Gyre. In nutrient amended microcosms containing MPn, a substrate-driven microbial succession occurred. Initially, the addition of glucose and nitrate resulted in a bloom of *Vibrionales* and a transcriptional profile dominated by glucose-specific PTS transport and polyhydroxyalkanoate biosynthesis. Transcripts associated with phosphorus (P) acquisition were also overrepresented and suggested that the addition of glucose and nitrate had driven the community to P depletion. At this point, a second community shift occurred characterized by the increase in C-P lyase containing microbes of the *Vibrionales* and *Rhodobacterales* orders. Transcripts associated with C-P lyase components were among the most highly expressed at the community level, and only C-P lyase clusters were recovered in a functional screen for MPn utilization, consistent with this pathway being responsible for the majority, if not all, of the methane accumulation we observed. Our results identify specific bacterioplankton taxa that can utilize MPn aerobically under conditions of P limitation using the C-P lyase pathway, and thereby elicit a significant increase in the dissolved methane concentration.

## Introduction

Methane (CH_4_) is an important component of the global carbon cycle and a potent greenhouse gas (Reeburgh, 2007). Despite the fact that the world's oceans are a significant source of CH_4_ contributing 1–4% of the annual global emissions (Karl et al., 2008), the origin of oceanic CH_4_ is poorly understood. While most of the water column is undersaturated for CH_4_, the surface concentration is supersaturated relative to atmospheric levels in much of the world's oceans, suggesting that a net source of CH_4_ exists there (Lamontagne et al., 1973; Rudd and Taylor, 1980; Tilbrook and Karl, 1995). Since the ocean surface is well-oxygenated and known methanogenenic pathways are inhibited by free oxygen (Rudd and Taylor, 1980), one prevalent hypothesis to explain CH_4_ supersaturation is the existence of methanogenesis within microanoxic aggregates. The ephemeral nature of anoxia in marine aggregates, however, might be predicted to limit the proliferation of slow-growing methanogens (Ploug et al., 1997). Recently it was hypothesized that microbial utilization of methylphosphonate (MPn) in P-limited surface waters might be a key source of aerobic CH_4_ production in the sea (Karl et al., 2008). MPn is the simplest of the phosphonate family of reduced P compounds which are characterized by the presence of a direct C-P bond instead of the more common C-O-P phosphate ester (Kittredge and Roberts, 1969). Nuclear magnetic resonance studies of high molecular weight DOP have shown that across oceans and throughout the water column, total phosphonates represent approximately one third of the dissolved organic P, and that phosphonate content decreases with depth in high molecular weight dissolved organic material (DOM) relative to C, indicating that phosphonates as a class of organic compounds are utilized by marine microorganisms (Clark et al., 1999; Kolowith et al., 2001). However, the chemical identity of marine phosphonates is only poorly understood. While compounds such as 2-aminoethylphosphonate (2-AEPn) (ciliatine) and 2-amino-phosphonopropionate (phosphonoalanine) had been found in many marine invertebrates (Horiguchi, 1984; Ternan et al., 1998), evidence of the occurrence of MPn had remained elusive. Recently, however, Metcalf et al. (2012) have shown that the abundant marine archaeon *Nitrosopumilus maritimus* encodes a novel pathway of MPn biosynthesis and produces an exopolysaccharide containing MPn esters (Metcalf et al., 2012). In addition, genes similar to those encoding MPn biosynthesis in *N. maritimus* were found to be relatively abundant in the planktonic marine microbial gene pool and were found in several scaffolds of the Global Ocean Survey database. Homologs of the MPn biosynthetic genes were also found in the abundant marine clade SAR11, suggesting that the concentration of MPn in seawater could be significant (Metcalf et al., 2012).

**Figure 1:**
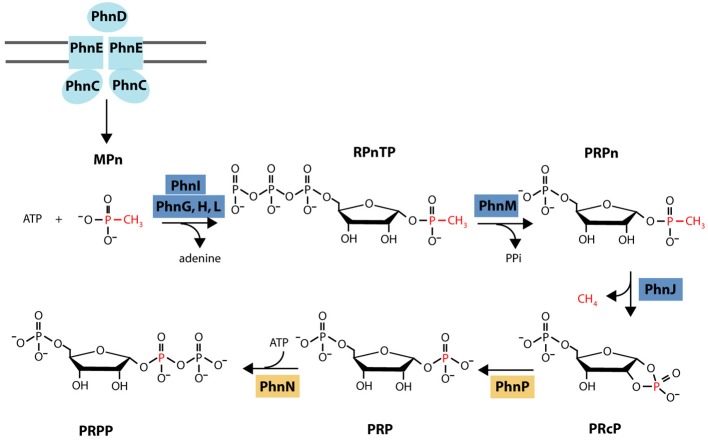
**Current biochemical model for MPn degradation**. MPn is transported into the cytoplasm by the Pn-specific ABC transporter system integrated by PhnC, PhnD, and PhnE. In the cytoplasm, PhnI catalyzes the conversion of MPn and ATP to α-D-ribose-1-methylphosphonate-5-triphosphate (RPnTP) and adenine in the presence of PhnG, H, and L. PhnM is a phosphatase that hydrolyzes RPnTP to form 5-phospho-a-D-ribosyl 1-alkylphosphonate (PRPn) which is the substrate of the C-P bond cleavage reaction catalyzed by PhnJ. C-P bond cleavage releases CH4 and 5-phospho-a-ribosyl-1,2-cyclic phosphate (PRcP). In the next two steps catalyzed by PhnP and PhnN, PRcP is converted to 5-phosphoa-D-ribosyl 1-diphosphate PRPP which is funneled into primary metabolism. Protein color coding as in gene cluster diagram (Figure 8). Diagram modified from (Zhang and Van Der Donk, 2012).

Microbial utilization of MPn is mediated by the C-P lyase pathway that hydrolyzes the C-P bond in a variety of aryl- and alkylphosphonates releasing the corresponding hydrocarbon (reviewed in Ternan et al., 1998; Quinn et al., 2007; White and Metcalf, 2007). In the case of MPn, C-P lyase activity yields CH_4_ that is released into the growth medium (Daughton et al., 1979). In *Escherichia coli*, where it is best characterized, the C-P lyase pathway is encoded in a 14 gene operon (*phnCDEFGHIJKLMNOP*) under the control of the P starvation Pho regulon (Metcalf and Wanner, 1991, 1993b; Wanner and Metcalf, 1992). Based on sequence and genetic analyses it was proposed that *phnCDE* encode a Pn-specific ABC transporter, *phnG* through *phnM* encode the minimal catalytic unit for C-P bond cleavage, and *phnF, phnN, phnO*, and *phnP* encode regulatory or accessory proteins. The biochemical steps involved and the identity of the oxidized P product remained uncharacterized until a recent series of papers that culminated with the C-P lyase activity finally being reproduced *in vitro* with MPn as the substrate (Hove-Jensen et al., 2003, 2010, 2011; Kamat et al., 2011, 2013). According to the current model (Figure 1), PhnI (in the presence of PhnG, H, and L) and PhnM catalyze the conversion of MPn to 5-phospho-α-D-ribosyl 1-alkylphosphonate (PRPn) which is the substrate of the C-P bond cleavage reaction catalyzed by PhnJ. C-P bond cleavage releases CH_4_ and 5-phospho-α-ribosyl-1,2-cyclic phosphate (PRcP). In the next two steps catalyzed by PhnP and PhnN, PRcP is converted to 5-phospho-α-D-ribosyl 1-diphosphate (PRPP) which is funneled into primary metabolism. While there appears to be considerable variability in the composition of the C-P lyase gene cluster among bacteria, all clusters include the *phnG-M* genes that are essential for C-P bond cleavage (Huang et al., 2005).

Since the presence of the C-P lyase genes in the marine cyanobacterium *Trichodesmium erythraeum* ISM101 was reported by Dyhrman et al. (2006), several marine bacterial strains containing C-P lyase clusters have been shown to grow in culture using MPn as the sole P source. These include *T. erythraeum* ISM101, *Ruegeria pomeyori* DSS-3, *Photobacterium angustum* S14, and *Planctomyces maris* DSM8797 (Martinez et al., 2010; White et al., 2010). Moreover, C-P lyase clusters are abundant in the genomes of sequenced marine strains and metagenomic libraries of surface waters of the Sargasso Sea (present in ~20% of bacteria) but less so at Station ALOHA in the North Pacific Subtropical Gyre (<1% of bacteria) (Martinez et al., 2010). Because one of the most prominent differences between the two ecosystems is that the surface dissolved inorganic phosphorus (DIP) concentrations are about an order of magnitude lower at the Sargasso Sea during the stratified summer months (Cavender-Bares et al., 2001), when the above samples were collected for sequencing, these results suggest that the ability to use Pn as a P source provides a competitive advantage in P-depleted waters (Coleman and Chisholm, 2010; Martinez et al., 2010). The high abundance of C-P lyase containing microbes in P-depleted waters could potentially result in significant MPn-driven CH_4_ production in these waters (Karl et al., 2008). Remarkably, the experiments by Karl et al. (2008) showed that even at Station ALOHA, incubation of seawater samples with MPn under amendment conditions leading to P-stress resulted in CH_4_ production indicating that despite the low abundance of C-P lyase clusters, the potential exists for MPn-driven CH_4_ production. To better characterize the microbes, biochemical and regulatory processes underlying this important process, we analyzed the changes in bacterioplankton community structure, gene content and gene expression that occurred after MPn amendment. Our results revealed a temporal succession of microbial taxa and metabolic process leading to the proliferation of initially low-abundance C-P lyase-containing microbes, and identified metabolic pathways and regulatory elements mediating the community response to nutrient addition.

## Results

### Experimental design, methane production, and cell counts

Previous experiments have shown that the natural surface microbial community at Station ALOHA in the North Pacific Subtropical Gyre rapidly consumed exogenous MPn and produced CH_4_ under aerobic conditions in 75 m water samples that had been amended with 100 μM glucose, 16 μM nitrate, and 1 μM MPn (referred to hereon as Glc+N+MPn) (Karl et al., 2008). In order to identify the microbes and gene functions mediating this process, we performed parallel metagenomic and metatranscriptomic analyses under similar enrichment conditions. Although additions of Glc+N were artificially high, we chose these conditions because they could promote the growth of indigenous MPn-utilizing microbes and thus facilitate the detection of MPn degradation genes normally found in low frequency in this environment (Karl et al., 2008; Martinez et al., 2010). Seawater was collected from 75-m depth below the mixed layer and above the deep chlorophyll maximum (Figure S1) and used immediately to set up 3 microcosm perturbation experiments as follows (Figure S2): B1, no amendment control; B2, Glc+N amendment with no added P source; and B3, Glc+N+MPn amendment. DNA samples were taken at the beginning and at the end of the experiment for genomic analyses. In addition, subsamples were taken at 12 h intervals for community RNA extraction and dissolved CH_4_ measurements. The concentration of P in the unamended water was 51 nmol l^−1^, within the typical range for the upper water column of the North Pacific Subtropical Gyre (Karl et al., 2008).

**Figure 2:**
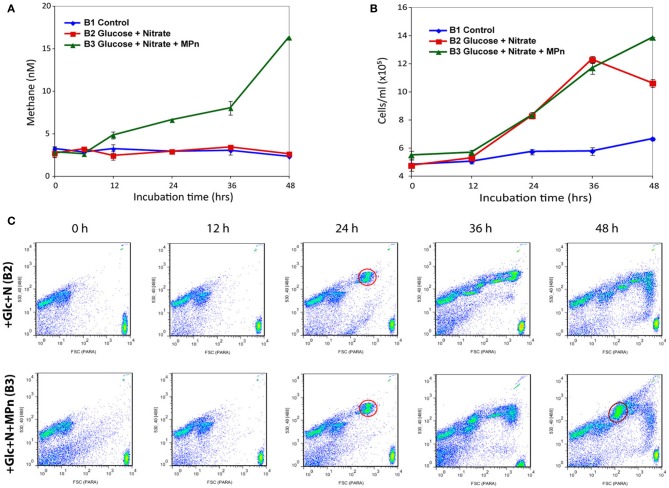
**CH_4_ accumulation and cell number dynamics during 48 h microcosm incubations**. Three microcosms containing 20 L of sea water were amended as follows: B1: no amendment control; B2: amended with 100 μM glucose and 16 μM nitrate; B3: amended with 100 μM glucose, 16 μM nitrate, and 1 μM MPn. Microcosms were incubated for 48 h and subsampled at 12 h intervals for CH_4_ measurements, flow cytometry, and community RNA. **(A)** CH_4_ accumulation in amended surface seawater samples. Dissolved methane concentration increases above background only in the Glc+N+MPn microcosm starting 12 h post amendment. **(B)** Flow cytometric counts of SYBR-stained microbial cells from the control and treatments. **(C)** Green fluorescence and forward scatter plots of treatment samples. The cell number increase is largely due to the appearance of distinct high DNA-content microbial populations. The first of such populations (highlighted in red) appeared in both treatments at 24 h and represented ~30% of the cells (2.6 × 10^5^ cells/ml) at that time point. The pattern remains similar between treatments at later time points with the exception of a unique large population that appears in the Glc+N+MPn microcosm 48 h after addition (circled in orange) that represents ~29% of the total cell counts at that time (~ 4 × 10^5^ cells/ml). Two micrometer fluorescent beads are at the bottom right of each panel.

The initial observed dissolved CH_4_ concentration in all three microcosms (2.6–3.2 nM) is typical of the upper water column in the North Pacific Subtropical Gyre (Karl et al., 2008). As was the case in the previous experiments, a significant increase in dissolved CH_4_ was observed only after Glc+N+MPn addition (B3) indicating that exogenous MPn was being converted to CH_4_ (Figure 2A). Elevated dissolved CH_4_ concentration compared to controls was first observed in B3 after 12 h of incubation and its concentration increased during the course of the experiment. A maximum CH_4_ concentration of 16.3 nM was achieved after 48 h of incubation representing an almost 6-fold increase over the initial concentration. These results are similar to those of previous experiments (Karl et al., 2008) although the observed CH_4_ concentrations were lower, likely because we did not use gas-tight vessels for the large volume microcosms.

While the cell numbers in the control microcosm increased only slightly during the course of the experiment, the number of cells increased significantly in both treatments starting 24 h after nutrient addition (Figure 2B). Flow cytometry analyses revealed that the increase was due to the appearance of specific populations of larger, high DNA-content cells which represented 30% of total cells with an approximate concentration of 2.6 × 10^5^ cells/ml (Figure 2C). Cell numbers and population distributions were identical in both amendments at the 24 and 36 h samples, suggesting that the observed changes were due to the Glc+N addition, while a distinct population unique to the Glc+N+MPn treatment appeared in the 48 h sample (~4 × 10^5^ cells/ml, or 29% of total cells). At the final 48 h time point, the cell counts are 155 and 210% higher than the control for the Glc+N and Glc+N+MPn treatments, respectively.

### The abundance of C-P lyase genes significantly increases after MPn amendment

The increase in CH_4_ concentration suggested the involvement of the C-P lyase pathway in MPn utilization. We analyzed the frequency of 6 genes encoding catalytic functions in this pathway (*phnG, phnH, phnI, phnJ, phnM*, and *phnN*) in community genomic DNA (gDNA) samples collected at the beginning and the end of the experiment (48 h). In addition to the C-P lyase genes, we included in the analyses other phosphonate-utilization genes previously identified in marine environments: *phnA*, encoding phosphonoacetate hydrolase, and *phnW, phnX, phnY*, and *phnZ* involved in 2-AEPn utilization (Quinn et al., 2007; Gilbert et al., 2009; Martinez et al., 2010). The *recA* gene was used as a reference single copy gene to normalize the abundance of the MPn degrading pathway genes. For each analyzed gene, abundance is expressed as the percentage of all bacteria containing the gene in question, based on the assumption that *recA* is present as a single copy gene in every genome. As it has been previously shown for surface waters in this region (Karl et al., 2008; Martinez et al., 2010), the frequency of *phn* genes was extremely low at the beginning of the experiment and after 48 h in the unamended microcosm (Table 1). Of the non C-P lyase *phn* genes analyzed, only the *phnW* and *phnX* encoding enzymes of the phosphonatase pathway for 2-AEPn utilization increased significantly in the Glc+N-amended microcosm (in ~28% of bacteria after 48 h). Phosphonatase gene abundance also increased but to a lesser extent in the Glc+N+MPn microcosm. Thus, the observed increased abundance of *phnW* and *phnX* does not appear to be MPn-driven, consistent with the fact that MPn is not a substrate for this pathway (Quinn et al., 2007). In contrast, the frequency of C-P lyase genes increased significantly only after Glc+N+MPn amendment. If we average abundance across all six analyzed C-P lyase genes, 40% of all bacteria are estimated to contain C-P lyase clusters after 48 h, compared to less than 1% at the beginning of the experiment. The vast majority of these hits are most similar to those of sequenced genomes of *Rhodobacterales* and *Vibrionales* (52% and 46% of all C-P lyase hits, respectively) (Table S1) suggesting that these two groups mediate the observed CH_4_ production. While the *Rhodobacterales phn* top hits were equally distributed across several species, 73 of the 74 identified *Vibrionales* sequences are most similar to those of a single sequenced strain, *V. nigripulchritudo* ATCC27043.

**Table 1:** **Abundance of*phn* genes in genomic DNA pyrosequencing libraries**.

**Substrate**	**Gene**	**TO**	**TF(48h)**		
		**No addition**	**No addition**	**Glc+N**	**Glc+N + MPn**
Alkyl-phosphonates	*phnG*	0	0	0	47
	*phnH*	0	0	0	15
	*phnI*	0	0	4	38
	*phnJ*	0	1	1	53
	*phnM*	0	0	2	67
	*phnN*	0	0	3	24
Phos- phonoacetate	*phnA*	2	6	4	7
2-Aminoethyl- phosphonate	*phnW*	6	5	25	5
	*phnX*	0	0	31	5
2-Aminoethyl- phosphonate	*phnY*	0	3	0	0
	*phnZ*	0	0	4	9
	*recA*	100	100	100	100

*Results expressed as % of microorganisms estimated to contain gene of interest, assuming recA is present in single copy in every bacterium (Martinez et al., 2010). Significance cut-off bit >50*.

### Microbial community structure

**Figure 3:**
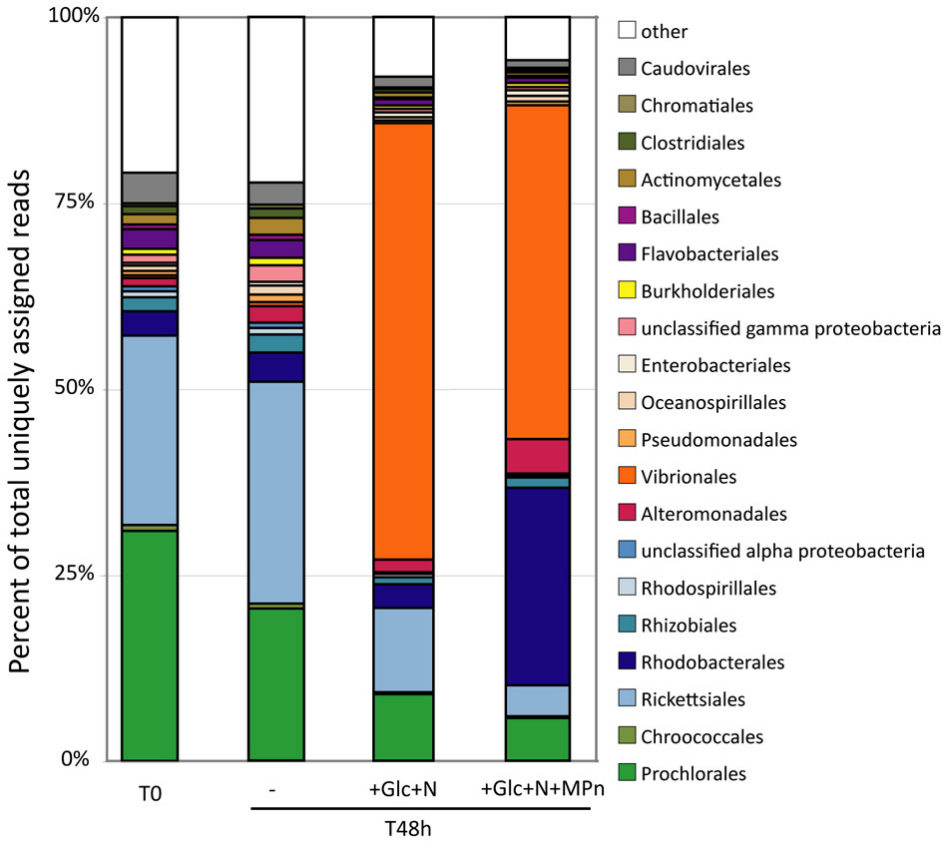
**Microbial population composition assessed by taxonomic classification of protein-coding metagenomic reads at the order level**. Only taxonomic groups that represent >1% of total assigned reads in at least one dataset have been included with all other groups binned together with unassigned reads. Abundance expressed as the percentage of reads assigned to each taxon divided by the total number of assigned reads in each database.

The changes in microbial community composition were assessed in more detail by taxonomic classification of protein-encoding metagenomic and metatranscriptomic reads. As expected, typically abundant planktonic bacterial taxa such as *Prochlorales* (*Prochlorococcus*) and *Ricketsiales* (*Pelagibacter*) were highly represented in the initial gDNA sample, and their abundance remained fairly constant over the course of the experiment in the unamended microcosm (Figure 3). In contrast, large shifts in community composition were observed in both the Glc+N and Glc+N+MPn treatments. In the Glc+N microcosm, the decrease in the relative abundance of *Prochlorales* and *Ricketsiales* was accompanied by a large increase in the abundance of Gammaproteobacteria of the *Vibrionales* order (59% of assigned reads, compared to less than 1% in the control). A dramatic increase in the abundance of *Vibrionales* (44%) was also observed in the Glc+N+MPn-amended microcosm compared to the no amendment control, but is accompanied in this case by a large increase in the abundance of Alphaproteobacteria of the *Rhodobacterales* order (27% of assigned reads vs. 4% in the controls), and to a much lesser extent, by an increase in *Alteromonadales* (4.6% vs. less than 2.2% in controls). Similar trends were observed for rRNA taxon abundance in metagenomic samples (Figure S3).

**Figure 4:**
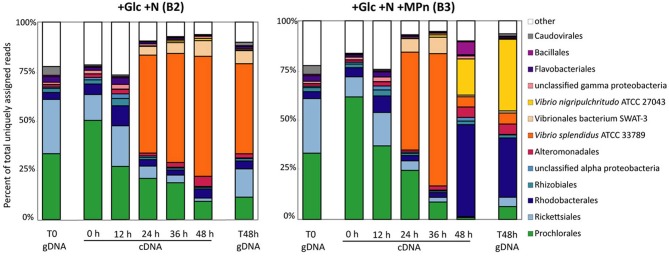
**Microbial population composition of amended microcosms assessed by taxonomic classification of protein-coding metatranscriptomic reads**. Abundance expressed as the percentage of reads assigned to each taxon divided by the total number of non-rRNA, non-sRNA assigned reads in each database. Taxonomic classification is shown at the order level except for the Vibrionales where read numbers for the thee main taxon id are shown. Only taxonomic groups that represent >3% of total assigned reads in at least one dataset have been included.

The taxonomic analysis of the intermediate metatranscriptomic samples revealed that up to the 36 h time point, both amended microcosms appeared to undergo parallel shifts in community composition with the large *Vibrionales* bloom taking place between 12 and 24 h, and continuing at the 36 h time point (Figure 4). This signal was due in the most part to one taxon, *V. splendidus* ATCC27043 (97% average id), for both amendments (Figures 4, S4). The fact that both microbial communities underwent this change suggests that the *V. splendidus* bloom was driven by the addition of Glc+N. While *V. splendidus* ATCC27043 remained the most abundant taxon in the 48 h cDNA and gDNA samples of the Glc+N-amended microcosm, the low levels of C-P lyase genes in the 48 h metagenomic samples (Table 1) indicate that this population does not have the capacity to utilize MPn.

The community composition profiles of the amended microcosms diverged only at the 48 h final time point when the large relative increase of *Rhodobacterales* was observed only in the Glc+N+MPn microcosm (Figure 4). At the taxon id level, these reads were equally distributed across many species with relatively low average similarity (78–82% amino acid identity) suggesting that the blooming *Rhodobacterales* have no close sequenced relatives (Figure S4). The relative abundance of *Bacillales* also increased in the 48 h transcriptome although to a lesser extent and many of those reads corresponded to hypothetical bacteriophage sequences which could compromise their taxonomic assignment. Finally, the relative abundance of *Vibrionales* decreased in the 48 h Glc+N+MPn transcriptome but more importantly, the abundance of *V. splendidus* sequences decreased significantly and a distinct *V. nigripulchritudo* ATCC 27043-like population (89% average id) became the most abundant taxon id at 48 h representing 15 and 32% of all assigned reads in the cDNA and gDNA samples, respectively (Figures 4, S4). Not only were *V. nigripuchritudo* and *Rhodobacterales* the same groups identified above in the taxonomic analysis of C-P lyase genes (Table S1), but combined they represented 45% of the assigned protein-coding reads at the metagenomic level, consistent with the estimated ~40% abundance of C-P lyase-containing microbes by the *recA* normalization method. Taken together, these results indicate that the Glc+N+MPn amendment led to a succession in the bacterioplankton population comprising an initial Glc+N-driven bloom of *V. splendidus* which was replaced by a MPn-driven increase in *Rhodobacterales* and *V. nigripulchritudo* populations. The apparent delay between the observed increase in dissolved CH_4_ concentration which started 12 h post- amendment (Figure 2), and the detection of MPn-driven population shift at 48 h could be the result of the limited sequencing depth and the lower abundance of these groups in the initial starting community.

It should also be noted that despite the increase in dissolved CH_4_ concentration observed in the Glc+N+MPn amended microcosm, we could not detect a significant increase in the relative proportion of reads assigned to any known aerobic CH_4_-oxidizing marine bacterial groups: (Methylococcales, Methylocystaceae, Verrucomicrobia, and *Methylophaga* spp.) (Hanson and Hanson, 1996; Dunfield et al., 2007; Neufeld et al., 2007).

### Functional gene expression trends in metatranscriptomic samples

In order to examine the functional processes underlying the community shifts observed in the amendments, bulk community cDNA reads were assigned to Kyoto Encyclopedia of Genes and Genomes (KEGG) functional categories and their relative abundance was compared across metatranscriptomes. For an initial assessment of the magnitude of functional gene expression changes, we calculated the fraction of KEGG orthologs whose abundance was significantly different (FDR-corrected *P*-value < 0.05) in the treatments compared to the unamended control at each time point. While this number was less than 5% in the 0 and 12 h cDNA samples, the percentage of functional KEGG orthologs with significant abundance changes relative to the unamended control increased significantly for both treatments in the 24 h sample (17%) and remained high throughout the treatment (Table S2). Not only were lower percentages of significant changes observed when the two amendments were compared directly against each other specially at the 24 h sample, but both amendments caused similar changes in the most abundant transcripts (Table S3) indicating that the shifts in functional gene expression were caused in part by the addition of Glc+N, in good agreement with the changes observed in taxonomic composition.

**Figure 5:**
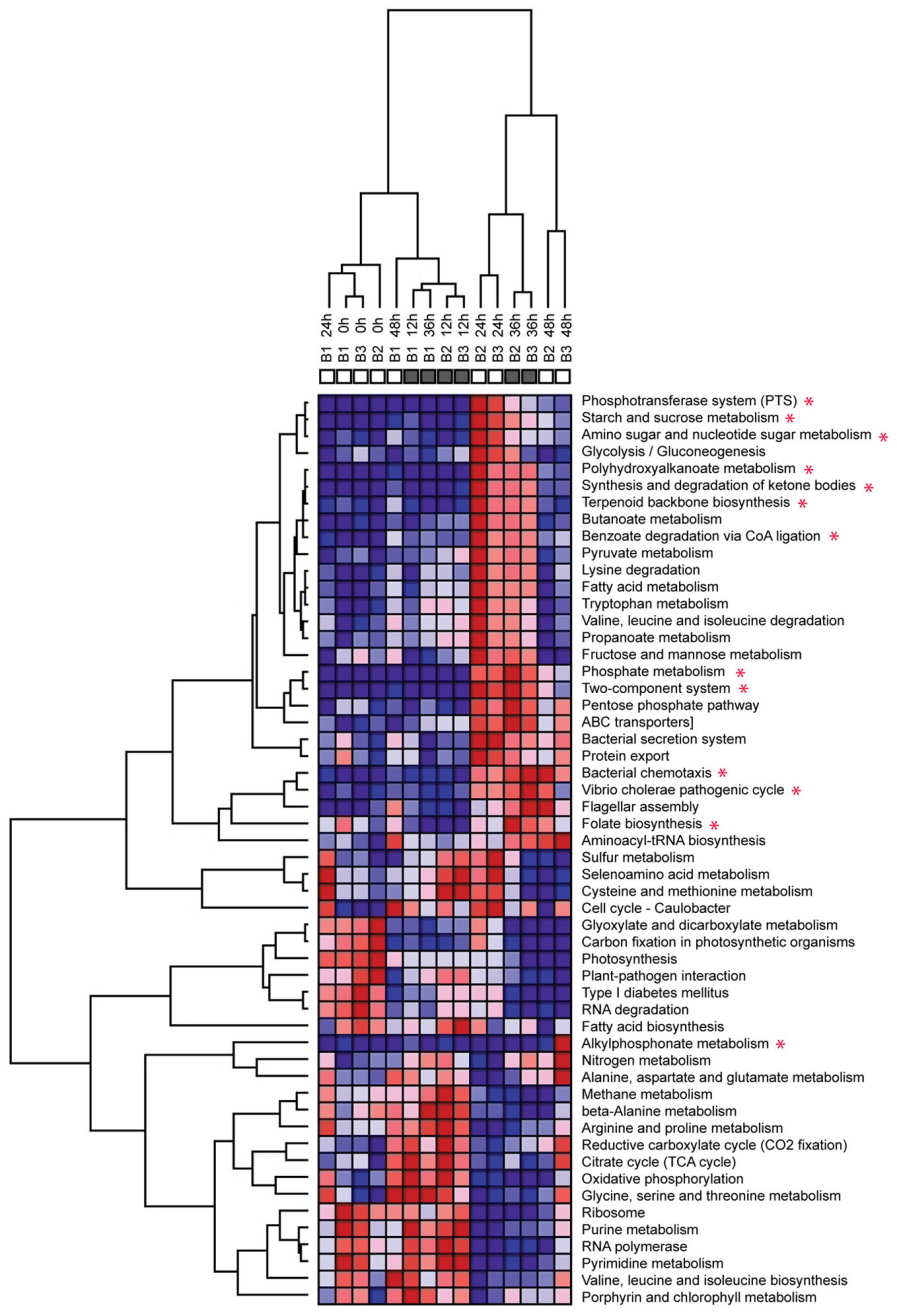
**Clustering of metatranscriptomics databases according to their KEGG pathway profiles**. Heatmap shows relative distribution of protein-coding reads matching KEGG pathways. Red = high abundance, blue = low abundance. Color scale is row (pathway) normalized. Only pathways representing more than 1% mapped reads in at least one dataset are included. Dendograms are based on hierarchical clustering of Pearson correlation coefficients for each pairwise comparison. KEGG pathways with at least 5-fold overrepresentation compared to the no addition control in at least one time point are marked with an asterisk. Pathway abundance data from Table S5.

**Figure 6:**
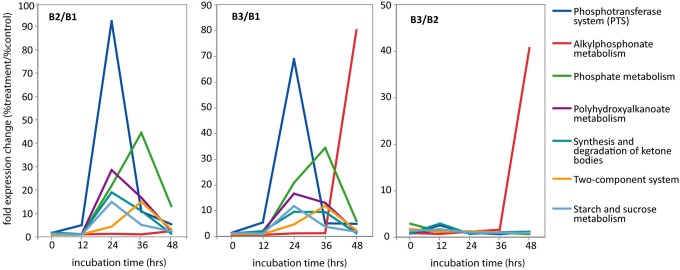
**Relative abundance of selected KEGG pathways in metatranscriptomic datasets**. For each time point, abundance is expressed as fold expression change relative to the no amendment control (% treatment/%control). **Left** panel (B2/B1): Glc+N/no amendment control; **Middle** panel (B3/B1): Glc+N+MPn/no amendment control; **Right** panel (B3/B2): Glc+N+MPn/Glc+N. For simplicity, only KEGG pathways with 10-fold or more overrepresentation in at least one time point are included. Complete data found in Table S6.

Clustering of the metatranscriptomes according to their KEGG pathway profiles results in two clearly distinct sample groups (Figure 5). The first group includes all the unamended control samples as well as the amended transcriptomes for the 0 and 12 h time points. Within this group, clustering appears largely due to the influence of the diel cycle, with high day time expression of photosynthetic and carbon fixation pathways and night time increase in oxidative phosphorylation and TCA cycle. This pattern is consistent with previously reported diel expression patterns in the surface waters (Zinser et al., 2009; Shi et al., 2010, 2011; Ottesen et al., 2011). In contrast, the metatranscriptomes of both amendments diverge from the control starting with the 24 h sample and form a separate amendment-driven cluster. Within this group, metatranscriptomes cluster by collection time and not by the specific amendment suggesting that a significant portion of the functional profile changes are driven by the Glc+N addition. Overall, after the 24 h time point the amendment metatranscriptomes are characterized by a large increase in the abundance of transcripts associated with organic carbon uptake and utilization, phosphate metabolism, and two-component regulatory systems among other changes (Figure 6). High expression of the alkylphosphonate metabolism pathway, which consist of the genes encoding C-P lyase components, is the major difference between the Glc+N and Glc+N+MPn transcriptomes. The most significant trends in community expression changes are highlighted below.

#### Phosphoenolpyruvate phosphotransferase system

Transcripts encoding components of the phosphoenolpyruvate phosphotransferase system (PTS) for sugar assimilation were very highly enriched in both amended transcriptomes (Figure 6). Expression of the PTS pathway peaked 24 h post-amendment with over 50-fold increase in relative expression compared to the unamended control. At 24 h, PTS transcripts constituted more than 3% of all assigned reads in both amendments compared to less than 0.1% in the B1 control (Table S5). Specifically, the most overrepresented transcripts included the glucose-specific components of the PTS (enzyme II subunits EIIA^gluc^ and EIIB^gluc^) as well as components of the general phosphotransfer cascade enzyme I (EI) and histidine protein (HPr) (Table S6). Thus, all components required for the sequential transfer of the phosphate group from phosphoenolpyruvate to EI, to HPr, to EII^gluc^, and finally to glucose as it is translocated across the membrane (Cordaro, 1976), are highly induced upon amendment indicating a shift toward glucose utilization at the 24 h time point in both amended microcosms. Transcripts for the sucrose (*scrA*) and N-acetylglucosamine-specific (*nagE*) EIIB components are also overrepresented in the amendment transcriptomes although to a lesser extent.

#### Polyhydroxyalkanoate biosynthesis

Concomitant with the increased expression of the glucose PTS transport system, we observed an overrepresentation of transcripts associated with the synthesis of polyhydroxyalkanoate (PHA). PHAs are storage lipid compounds that accumulate when an essential nutrient becomes limiting under carbon excess conditions and serve as carbon and energy storage for maintenance of metabolism during starvation and growth resumption in many bacteria (Rehm and Steinbuchel, 1999; Waltermann and Steinbuchel, 2005). As was the case with PTS, expression of the PHA synthesis pathway peaked for both Glc+N and Glc+N+MPn transcriptomes 24 h post-amendment (28 and 16-fold overrepresentation compared to control, respectively), and decreased gradually thereafter (Figure 6). Transcripts for all three enzymes required for PHA biosynthesis, acetyl-CoA acetyltransferase (*phbA*), acetoacetyl-CoA reductase (*phbB*), and polyhydroxyalkanoate polymerase (*phbC*), were very significantly enriched, ranging from 10- to more than 600-fold over control at 24 h (Table S6). The large number of transcripts for the acetoacetyl-CoA reductase observed at 24 h was enough to drive by itself an apparent increase of several other KEGG pathways including those for synthesis and degradation of ketone bodies, benzoate degradation, and terpenoid backbone biosynthesis. Because no other transcripts in those pathways showed similar increase in abundance, they will not be discussed further.

As observed for the PTS pathway, the majority of the transcripts associated with PHA biosynthesis are most similar to those of *Vibrionales*, in particular *V. splendidus*, which was the dominant taxon at this time. Accumulation of poly-β-hydroxybutyrate in vibrios has been previously observed in culture during the growth phase and when undergoing P starvation in the presence of excess glucose (Mårdén et al., 1985; Malmcrona-Friberg et al., 1986).

#### Other carbohydrate utilization pathways

Several additional KEGG pathways involved in carbohydrate metabolism are also overrepresented in the two amendments relative to control, most significantly the starch and sucrose metabolism and amino sugar and nucleotide sugar metabolism (Table S6). While at 24 h the transcripts for the glucose-specific components of the PTS described above dominate the signal in these pathways, transcripts associated with the utilization of chitin, starch and sucrose are overrepresented in the 48 h transcriptomes suggesting a shift toward more complex substrate utilization at the final time point for both amendments.

#### Phosphate metabolism

Transcripts associated with P acquisition systems were highly overrepresented in the transcriptomes of both amended microcosms indicating that the addition of Glc+N drove the microbial community into P starvation (Figure 6). The relative increase is observed starting at 24 h but it is at its maximum 48 h post-amendment (44 and 34-fold increase after Glc+N and Glc+N+MPn addition, respectively). The most enriched transcripts include the phosphate-binding (*pstS*), permease (*pstC*), and ATP-binding (*pstB*) components of the high affinity phosphate-specific ABC transporter (Table S6), which is known to be induced under P starvation in many organisms (Scanlan et al., 1993; Wanner, 1993; Dyhrman and Haley, 2006; Martiny et al., 2006; Dyhrman et al., 2007). The periplasmic phosphate-binding component *pstS* in particular is the most highly expressed KEGG ortholog in both amendments at 24 and 36 h, and at 48 h in the Glc+N microcosm (Table S3). Also highly enriched were transcripts encoding alkaline phosphatases *phoA* and *phoX* which mediate utilization of dissolved organic P compounds under low phosphate conditions (Sebastian and Ammerman, 2009; Duhamel et al., 2010) again indicating a shift toward P-limitation after Glc+N addition. Also enriched were the phosphate regulon two-component sensor (*phoR*) and transcriptional regulator (*phoB*).

#### Nitrogen metabolism

Several transcripts associated with nitrogen metabolism were overrepresented in both amendments. In particular, the NADH-dependent catalytic subunit of the assimilatory nitrate reductase (*nasA*), and the large (*nirB*) and small (*nirD*) subunits of the NAD(P)H-dependent nitrite reductase were significantly enriched 48 h post-amendment reflecting the assimilation of the allochthonous nitrate by sequential reduction to nitrite and ammonia (Moreno-Vivian et al., 1999).

#### Two-component systems, chemotaxis, and flagellar synthesis

Another dramatic change in expression profile occurring in both amendment transcriptomes and thus, induced by the Glc+N addition, is the overrepresentation of transcripts encoding two-component sensor systems starting 24 h post-amendment (Figure 6). Specifically, both components of the phosphate-sensor system (*phoR/phoB*) were overrepresented, as well as those associated with carbon storage regulation (*barA/uvrY*), autoinducer 2 sensing (*luxP/luxQ/luxO*), flagellar synthesis (*flrA/flrB*), stress response (*rstB/rstA*), redox state sensing (*arcA*), and C4-dicarboxylate (*dctB/dctD*) (Table S6). In addition, the chemotaxis sensor kinase (*cheA*) and response regulator components (*cheV* and *cheY*) were very significantly enriched (up to 35-fold compared to control) as were the other components of the bacterial chemotaxis system controlling flagellar rotation (*mcp, cheR, cheB*, and *cheW*). Also significant were the enrichment of the flagellar-specific sigma factor (*fliA*) as well as many flagellar components and assembly proteins (Table S6). This pattern of two-component system transcript enrichment, similar to that observed after HMW DOM addition (McCarren et al., 2010), indicates a shift toward an “opportunistic” metabolic profile capable of sensing and reacting quickly to nutrient fluctuations (Polz et al., 2006; Lauro et al., 2009; Stocker, 2012).

#### ABC transporters

The amendments also resulted in an enrichment of transcripts associated with ABC transporters involved in ATP-driven nutrient uptake, but while some of those changes are common to both amendments, others are amendment specific. The main common ABC transporter increase was that of the phosphate-specific transporter discussed above (*pstS/pstC/pstA*) but an enrichment of transcripts encoding components of the maltose-specific (*malE/malF/malG*) and glycerol-3-phosphate-specific transporters (*ugpE/ugpA/ugpE*) are also observed (Table S6). The Glc+N+MPn transcriptome presented unique ABC transporter enrichments at the final 48 h time point for xylose (*xylF/xylH*) and phosphonate transport (*phnD/phnE/phnC*). These transcriptional changes are likely to be directly or indirectly MPn-driven. The phosphonate transporter expression changes are discussed in detail below.

#### Other changes

The lipoprotein NlpD exhibited one of the largest fold increases in both amendments starting at 24 h post addition (Table S6). NlpD is a component of the cell division machinery that regulates the activity of peptidoglycan-degrading amidases at the cytokinetic ring (Uehara et al., 2010) and thus its increase in expression is consistent with the observed bloom occurring in response to Glc+N addition at 24 h (Figure 2). A similar pattern was observed for *tatB* and *tatA* which encode components of the sec-independent twin-arginine translocation pathway (Lee et al., 2006). Interestingly secretion of the alkaline phosphatase PhoX, highly induced upon amendment, has been shown to take place by the TAT pathway (Monds et al., 2006).

#### Small RNAs

**Figure 7:**
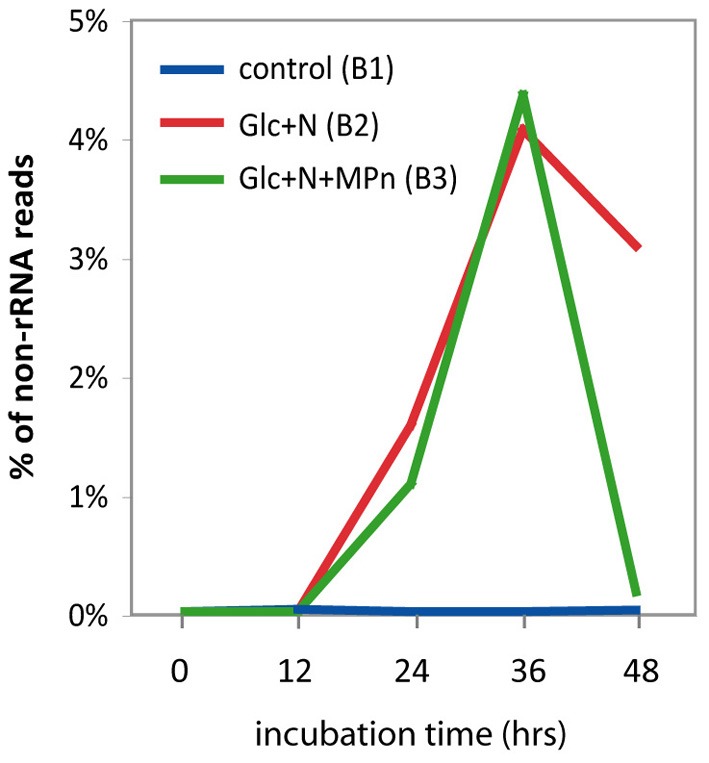
**Relative abundance of CsrB/RsmB sRNA in metatranscriptomic datasets**. Abundance is expressed as the percentage of non-rRNA sequences matching CsrB with a significance cut off of *b* > 50.

Since it is known that small regulatory RNAs (sRNAs) are abundant in marine microbial transcriptomic data (Shi et al., 2009) we examined the abundance of known sRNAs in our samples in an attempt to unveil possible sRNA-mediated regulatory mechanisms underlying the massive changes observed in the metabolic profile of the amended microcosms. Based on the Rfam 10.0 database (Gardner et al., 2009), we identified several sRNA families that were overrepresented after nutrient amendment (Table S7). The most significant change was the enrichment in sRNAs of the *csrB/rsmB* family which were barely detectable in the control transcriptome but increased significantly after 24 h and constituted more than 4% of the non rRNA reads at the 36 h time point for both amendments (Figure 7). Not surprisingly, the vast majority of the *csrB* reads were most similar to those of *V. splendidus*, the major taxon blooming 24 h after Glc+N addition. The CsrB (for carbon storage regulator) or RsmB (for repressor of stationary-phase metabolites) sRNAs use molecular mimicry to sequester multiple copies of the regulatory protein CsrA (or RsmA) away from their cognate mRNAs and thus alter their translation or turnover (reviewed in Romeo et al., 2013). Originally described in *E. coli* as a regulator of glycogen biosynthesis, CsrA is known to be a pleitropic regulator that, depending on the organism, controls central carbon flux, cell motility, biofilm formation, production of extracellular products, quorum sensing or pathogenesis (Babitzke and Romeo, 2007). Transcripts associated with several of these pathways were significantly enriched in the amended transcriptomes, as were the transcripts for the *BarA/UvrY* two-component system that regulates CsrB expression in response to unknown metabolic signals, suggesting that CsrB may play an important role in regulating gene expression in response to nutrient addition in our experiments. Other sRNAs whose abundance increases significantly in response to Glc+N addition include among others the glycine and lysine riboswitches (Table S7) again suggesting that sRNAs play a significant role in the response to environmental stimuli by bacterioplankton communities.

#### Alkylphosphonate metabolism

The major difference at the functional level between the two amendments is the overrepresentation of transcripts associated with the alkylphosphonate metabolism in the 48 h transcriptome of the Glc+N+MPn microcosm (Figure 6). Specifically, alkylphosphonate metabolism expression was 80-fold and 40-fold more abundant in this metatranscriptome than in the no amendment or Glc+N control, respectively. The enrichment is observed for all members of the C-P lyase pathway with the exception of *phnO* which was not detected in our sample (Table S6). PhnO is an alkylphosphonate N-acetyltransferase recently implicated in utilization or detoxification of certain amino-phosphonates but in *E. coli* it is not required for MPn degradation *in vivo* or *in vitro* with purified enzymes (Errey and Blanchard, 2006; Hove-Jensen et al., 2012). Thus, all components known to be necessary for MPn utilization, including the phosphonate-specific ABC transporter (*pnhCDE*), transcriptional regulator (*phnF*), as well as catalytic units (*phnGHIJKLMNP*) (Metcalf and Wanner, 1991, 1993a,b; Yakovleva et al., 1998), were highly enriched in the MPn 48 h transcriptome. The extent of the enrichment was high, 6- to 250-fold depending on the ortholog (Table S6). One of them, *phnM*, was among the top 10 most abundant orthologs in the 48 h MPn-containing sample (0.5% of all reads mapped to KEGG but undetectable in the no-amendment control, Table S3).

The fact that no significant C-P lyase expression was detected at the earlier time points despite the fact that dissolved CH_4_ concentration was observed starting 12 h post-amendment is likely to be a combination of the low sequencing depth and the very low relative abundance of C-P lyase-containing microbes in the original community (Table 1) which is in agreement with previous analyses in this environment (Karl et al., 2008; Martinez et al., 2010).

#### CH_4_ utilization

Given the significant increase in dissolved CH_4_ concentration observed in the Glc+N+MPn-amended microcosm, one might expect to see an increase in transcripts associated with CH_4_ utilization. Reads encoding the particulate (*pmoABC*) or soluble (*mmoBCDXYZ*) methane monooxygenases catalyzing the first step in aerobic oxidation of CH_4_ (Hanson and Hanson, 1996) were not detected in either the metatranscriptome or metagenome. Similarly, there was no increase in the abundance of transcripts encoding the methyl-coenzyme M reductase (*mcrABCD*) or the sulfite reductase (*dsrAB*) which have been used as markers for anaerobic methanotrophic archaea or their associated sulfate reducing bacteria, respectively (Havelsrud et al., 2011). These results, consistent with the absence of abundance shifts in known methanotrophic groups in the taxonomic profile, suggest that methanotrophy was not significantly induced under our experimental conditions and sequencing depth, most likely due to the short time course of the incubation, and competition for nutrients by other bacterial heterotrophs.

### Functional screening for MPn utilization pathways

We performed a functional screen for MPn degradation pathways in order to gain a better understanding of the phylogenetic origin and gene composition of the C-P lyase clusters identified through our metatranscriptomic analysis, and to identify any other existing MPn degradation pathways involving uncharacterized enzymes. This approach, which does not rely on any *a priori* sequence information has been successful in identifying novel Pn degradation pathways in a marine microbial metagenomic library (Martinez et al., 2010).

**Figure 8:**
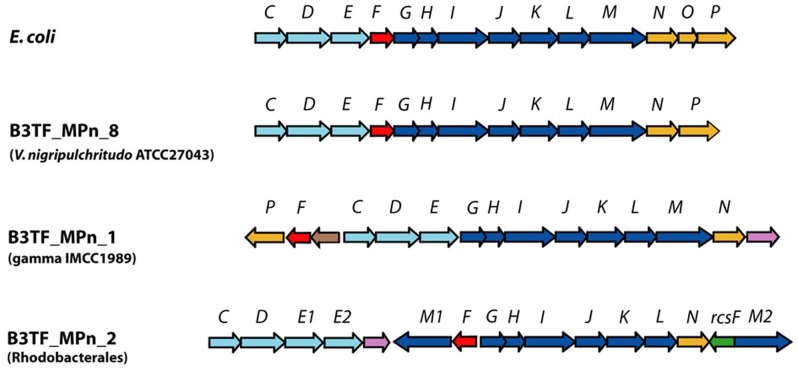
**Schematic representation of C-P lyase gene clusters in *E. coli* and environmental fosmids identified in the MPn utilization functional screen of the B3_TF library**. Genes are marked according to their assigned function as follows: putative ABC transporter components (light blue), catalytic components required for MPn degradation (dark blue), regulatory (red) and accessory proteins (orange). New genes are marked as follows: green, *rcsF* phosphoesterase probably analogous to *phnP*; brown: putative acyl-CoA N-acetyltransferase; purple: putative transferase.

We prepared a fosmid library (~47,000 clones with 40 kb average insert size for a total of ~1.9 Gb content) using the 48 h metagenomic DNA from the Glc+N+MPn microcosm and screened it for clones that could complement *E. coli* BW16787trfA (Martinez et al., 2010) for growth on MPn as the sole P source. This strain has a partial deletion in the *phn* operon (Δ*phnHIJKLMNOP*) that renders it incapable of growing on Pn, while still maintaining a functional Pn transporter encoded by *phnCDE*, the first three genes of the operon (Figure 8). After de-replication, we found that the 25 clones that allowed growth on MPn represented overlapping clones of 3 distinct fragments. A representative of each group was fully sequenced and all three were found to encode C-P lyase clusters (Table S8). Based on the phylogenetic affiliation of the best blast hits for each of the predicted encoded proteins, B3_TF_MPn_2 (19 clones) belongs to an Alphaproteobacterium of the *Rhodobacteraceae* family (66% average protein identity across several *Rhodobacteraceae* genomes) while B3_TF_MPn_8 (4 clones) is highly similar to *V. nigripulchritudo* ATCC27043 (82% average protein identity across the entire fosmid). Finally, the origin of the least abundant hit (B3_TF_MPn_1, 2 clones) is likely to be a Gammaproteobacterium of the *Alteromonadales* order, with the oligotrophic marine Gammaproteobacterium IMCC1989 (Jang et al., 2011) as the best hit for 12 of its 30 predicted proteins. The phylogenetic origin of the MPn screen hits and approximate relative abundance were consistent with the community changes observed in the final 48 h time point of the MPn amendment (see Figure 3). In addition, the vast majority (80%) of the C-P lyase gDNA reads identified (Table 1) could be assigned to the C-P lyase clusters in B3_TF_MPn2 (*Rhodobacterarales)* and B3_TF_MPn_8 (*V. nigripulchitudo*) using a stringent 95% nucleotide identity cutoff indicating that these two clusters represent the majority of the C-P lyase genes in our samples. Furthermore, the mean expression ratio (RNA/DNA) of the C-P lyase cluster genes in the final 48 h sample is more than 10 times higher than that of non-C-P lyase genes in the same fosmids (Figure S5) underscoring the important role of the C-P lyase in MPn metabolism under our conditions.

All three marine C-P lyase clusters identified contained all the genes required for MPn cleavage in *E. coli* (Figure 1). They include those encoding the phosphonate-specific ABC transporter system (*phnC, phnD, and phnE*), the regulatory gene *phnF*, and *phnG* through *phnM* which encode required catalytic functions to release CH_4_ from MPn (Metcalf and Wanner, 1991, 1993a,b; Yakovleva et al., 1998). A few significant differences with the *E. coli phn* cluster were observed. For example, *phnP* which encodes a phosphoribosyl cyclic diesterase that cleaves the product of the C-P bond hydrolysis reaction, 5-phospho-α-D-ribosyl-1,2-cyclyc phosphate (PRcP) (Figure 1) (He et al., 2011; Hove-Jensen et al., 2011), is not present in B3_TF_MPn_2. However, an unrelated protein also belonging to the 2H phophoesterase superfamily of cyclic nucleotide phosphodiesterases (Mazumder et al., 2002) is encoded in this cluster (green arrow in Figure 8) as well as in other C-P lyase clusters of sequenced microbes which also lack *phnP* (Huang et al., 2005). Therefore, it is likely that this protein, sometimes referred to as RcsF, plays the same role in MPn degradation as PhnP by hydrolyzing the cyclic phosphate in PRcP thus channeling the P in MPn to primary metabolism.

Another important characteristic of these clusters is that all lack a *phnO* gene which encodes a aminoalkylphosphonate N-acetyltransferase that in *E. coli* is required for catabolism and detoxification of 1-aminoalkylphosphonic acids (Errey and Blanchard, 2006; Hove-Jensen et al., 2012). Therefore, we predicted that none of the 3 marine clusters would enable utilization of 1-aminoalkylphosphonates. To test this hypothesis, we analyzed the Pn specificity of *E.coli* strains expressing each of the three clusters (Table 2) and found that while all of them allow the use of MPn, ethylphosphonate, 3-phosphonpropionate, 2-aminoethylphosphonate, and 3-aminophosphonopropionate as the sole P source, they were not able to grow on aminomethylphosphonate or 1-aminoethyphosphonate, consistent with the lack of *phnO*.

**Table 2:** **Phosphonate specificity of MPn clones**.

	**no P**	**MPn**	**AMPn**	**2AEPn**	**1AEPn**	**Epn**	**PnAc**	**PnAla**	**3PnP**	**3APPn**	**HPt**	**Pt**	**Pi**
CC1FOS	−	−	−	−	−	−	−	−	−	−	−	−	+
B3_TF_Mpn_1	−	+	−	+	−	+	+	+	+	+	−	+	+
B3_TF_Mpn_2	−	+	−	+	−	+	−	−	+	+	−	−	+
B3_TF_Mpn_8	−	+	−	+	−	+	+	−	+	+	−	+	+

*BW16787 harboring CC1FOS (negative control), B3_TF_Mpn_1, B3_TF_Mpn_2, and B3_TF_Mpn_8, were grown in liquid MOPS glycerol cultures with 0.3 mM of the following P sources: methylphosphonate, MPn; aminomethylphosphonate, AMPn; 2-aminoethylphosphonate, 2AEPn; 1-aminoethylphosphonate, 1AEPn; ethylphosphonate, EPn; phosphonoacetate, PnAc; phosphonoalanine, PnAla; 3-phosphonopropionate, 3PnP; 3-aminopropylphosphonate, 3APPn; hypophosphite, HPt; phosphite, Pt; and phosphate, Pi. Optical density at 600 nm was used to evaluate growth after 6 days of incubation*.

Finally, several genes encoding proteins of unknown function are found embedded in the new marine C-P lyase clusters and in the clusters of related sequenced marine genomes. These include a second putative *phnM*-like metal-dependent hydrolase and a second *phnE* permease component of the ABC transporter in B3_TF_MPn_2 (*phnM1* and *phnE2* in Figure 8), a putative acyl-CoA N-acetyltransferase of the GNAT family in B3_TF_MPn_ 2 (brown in Figure 8), and a putative transferase of the hexapeptide repeat family in B3_TF_MPn_1 and 2 (purple in Figure 8). Although the exact role of these proteins in Pn utilization remains to be defined, they may mediate the utilization of particular Pn substrates found in the marine environment. Nevertheless, the broad substrate specificity of all three marine clusters implies that the microbes that were enriched after MPn amendment could also be involved in the degradation of other Pns present in the marine environment such as 2-AEPn, found as a side group of glycoproteins and exopolysaccharides and in the polar head group of phosphonolipids of many marine organisms (Horiguchi, 1984), or the recently described MPn esters produced by the abundant marine archaeon *Nitrosopumilus maritimus* (Metcalf et al., 2012).

No non-C-P lyase MPn utilization pathways were identified in our functional screens. Although we cannot exclude the possibility that other MPn pathways may exist in the library but were not found because of possible expression problems in the heterologous host, the fact that we recovered multiple overlapping C-P lyase clusters from the major taxonomic groups enriched during the MPn-amendment experiment suggests that the C-P lyase pathway was the major metabolic pathway responsible for MPn utilization in this experiment.

## Discussion

One of the major challenges of microbial ecology is to describe, quantify and model the activities of microbes in global biogeochemical cycles. Because the oceans represent 1–4% of global CH_4_ emissions, the qualitative and quantitative contributions of microbes to the upper ocean's CH_4_ cycle is of significant interest. It has been hypothesized that aerobic utilization of MPn in P-limited waters could contribute significantly to the upper ocean's CH_4_ supersaturation (Karl et al., 2008), yet very little is known about the microbes, metabolic pathways and environmental conditions involved. In this study, short term incubation of natural bacterial populations collected in the oligotrophic North Pacific Subtropical Gyre under conditions leading to CH_4_ accumulation revealed a temporal succession of microbial taxa and metabolic processes. Initially, the addition of Glc+N led to a large bloom of a population highly similar to sequenced *V. splendidus* strains. Protein encoding transcripts associated with this taxon increased from less than 1% to more than 42% of the metatranscriptome uniquely assigned reads in a12 h period demonstrating the superior ability of these organisms to respond quickly to elevated nutrients, a typical trait of organisms with a trophic strategy referred to as opportunistic (Polz et al., 2006; Lauro et al., 2009). In contrast to microbes adapted to grow optimally under oligotrophic conditions, opportunistic microbes grow optimally under high nutrient conditions, typically have multiple rRNA operons, maintain elevated numbers of ribosomes during starvation, and possess a wide variety of nutrient sensing and metabolic pathways (Kramer and Singleton, 1992; Flardh and Kjelleberg, 1994; Polz et al., 2006; Lauro et al., 2009). Our transcriptomic data provide an opportunity to analyze in detail the regulatory and metabolic processes underlying such quick response to nutrient enrichment. Addition of Glc+N led to the elevated representation of transcripts associated with the Glc-specific PTS system that mediates Glc uptake and of those encoding enzymes to synthesize PHAs from acetate, the product of glycolysis. Given that the original concentration of DIP in the seawater was 51 nM, the growth elicited by Glc+N addition (600 μM C and 16 μM N, respectively), is predicted to quickly lead to P limitation provided all other required trace elements and organic cofactors (i.e., vitamins) are present in excess. Our data clearly show an enrichment of transcripts associated with P acquisition such as the phosphate-specific ABC transporter, the alkaline phosphatases *phoX* and *phoA*, as well as *phoB* and *phoR*, the master regulators of the Pho operon. While some of these transcripts appear to originate from other members of the community, the majority of the signal corresponds to *V. splendidus* underscoring the importance of P acquisition to support its population bloom. In contrast, the metagenomic data from the final community in the Glc+N microcosm, were *V. splendidus* reads constitute a large fraction of the sample, indicate that this population does not harbor a C-P lyase gene cluster, and thus is presumably not able to use MPn as a P source.

Accumulation of poly-β-hydroxybutyrate has been previously observed in culture for vibrios grown in the presence of excess glucose (Mårdén et al., 1985; Malmcrona-Friberg et al., 1986). Ovreas et al. (2003) reported that a large-celled *V. splendidus* population filled with C-rich granules became dominant when a natural marine bacterial community was forced to nutrient-limited growth by adding excess glucose (Ovreas et al., 2003). These authors suggested that the success of the large bacteria in glucose-replete treatments is not a consequence of their superior glucose utilization abilities, but rather of their advantage competing for the limiting nutrient resulting from the combination of a large surface for nutrient uptake and low cellular content of the limiting nutrient made possible thanks to the accumulation of C-rich storage granules. In culture, *V. splendidus* experiences a transition from small coccoid cells in C-limiting cultures, to large cells containing C-rich granules under P-limiting conditions (Lovdal et al., 2008). The large C-rich cells under P-limiting conditions exhibited high alkaline phosphate activity and a measured affinity for orthophosphate approaching the theoretical maximum predicted by diffusion-limited uptake (where every phosphate molecule that enters in contact with the cell surface would be taken up) indicating that the large size did not compromise their ability to compete for limiting P. The very high levels of expression of PHA biosynthetic genes, periplasmic alkaline phosphatases and phosphate ABC-transporter components, particularly the periplasmic phosphate-binding protein PtsS observed in our transcriptomic results, as well as the simultaneous appearance of large size cell populations observed by flow cytometry are consistent with this hypothesis. The ability of *V. splendidus* to use non-limiting organic C to synthesize PHA and thus augment its cell size without increasing its requirement for P or N may provide a competitive advantage for mineral nutrient acquisition under excess C and explain why it was the minor *V. splendidus*-like component of the bacterial community the group that dominated the glucose-driven initial community shift in the amendments, instead of the *Alteromonadales* observed after high molecular weight dissolved organic matter addition (McCarren et al., 2010).

Following the initial fast response to Glc+N addition observed at 24 h, our transcriptomic results indicated that as the initial bloom progresses, acquisition of other nutrients becomes important. For example, we observed enrichment of transcripts associated with glycerol-3-phosphate, maltose, and spermidine-specific ABC transporters, as well as the extracellular chitinase and other genes involved in the uptake and metabolism of the resulting N-acetylglucosamine monomers. This ability to access a variety of nutrients is characteristic of opportunistic microbes (Polz et al., 2006; Lauro et al., 2009). Finally, we observed an enrichment of transcripts associated with flagellar biosynthesis, motility, and chemotaxis. The chemotactic response that enables marine bacteria to access and exploit short-lived nutrient patches, is also characteristic of the opportunistic lifestyle (Polz et al., 2006; Stocker et al., 2008).

Once the microbial community was driven into P starvation after the Glc+N addition, a second population shift took place in the microcosm amended with MPn characterized by the enrichment in microbes containing C-P lyase gene clusters. While the low abundance of Pn utilization genes is consistent with previous observations for metagenomic samples from this area (Karl et al., 2008; Martinez et al., 2010), the high frequency of C-P lyase-containing microbes observed at the end of the experiment is similar to that previously reported for the surface waters of the Sargasso Sea during the stratified summer months when phosphate concentrations are one to two orders of magnitude lower than in the NPSG (Wu et al., 2000; Cavender-Bares et al., 2001; Martinez et al., 2010). Taken together these results indicate that under conditions of P limitation, the capacity to compete for available Pn became a critical component shaping the microbial community.

In the Sargasso Sea the majority of the C-P lyase reads were most similar to genes from *Candidatus Pelagibacter sp*. HTCC7211 (Martinez et al., 2010), a highly abundant member of the SAR11 clade of Alphaproteobacteria adapted to oligotrophic conditions (Stingl et al., 2007). In contrast, the MPn-degrading microbes enriched in our experiment belonged to the *Vibrionales* and *Rhodobacterales* orders which were only minor components of the Sargasso Sea signal. The success of these groups under our enrichment conditions is likely to be due to their ability to proliferate quickly in response to Glc additions. Similar to vibrios, members of the *Roseobacter* clade have an opportunistic lifestyle and harbor an enormous diversity of metabolic activities (Moran et al., 2004, 2007). Given that members of this group have been reported to dominate microbial glucose uptake in nutrient enrichment experiments (Alonso and Pernthaler, 2006) and the genomes of sequenced *Rhodobacterales* often contain C-P lyase gene clusters [25/32 sequenced genomes (Newton et al., 2010)], it is not surprising that the *Rhodobacterales* became one of the two dominant groups in our Glc+N+MPn experiment.

Three functional C-P lyase clusters were recovered from the metagenomic DNA and we were able to show that they allow growth using various Pn in addition to MPn as the sole P source. While there were differences in substrate specificity among the three clusters probably mediated in part by the novel uncharacterized proteins present, all of them elicited the utilization of 2-AEPn which is believed to be the most abundant Pn in the marine environment (Horiguchi, 1984) suggesting that the marine microbes they came from could be able to access a variety of Pn compounds as their P source. The importance of the recovered C-P lyase pathways in our experiment was underscored by the high expression ratio of its components in the metagenomic and metatranscriptomic samples. This was not the case for the phosphonatase *phnW* and *phnX* genes encoding enzymes for 2-AEPn which were highly enriched in the Glc+N microcosm but whose expression was below detection in the metatranscriptomic samples. Their lack of expression despite the observed induction of other P acquisition genes is consistent with the observation that phosphonatase genes are often induced by their substrate and not by P starvation as is the case for C-P lyase (Ternan et al., 1998; Ternan and Quinn, 1998; Quinn et al., 2007; White and Metcalf, 2007).

Taken together our results confirm the hypothesis of Karl et al. (2008) that aerobic utilization of MPn by C-P lyase containing microbes can lead to CH_4_ supersaturation under P-stress conditions. How significant are the taxa and pathways identified in this study to the overall CH_4_ cycle? Even in the oligotrophic surface waters of the NPSG where the C-P lyase containing groups were not abundant (<1% total cell population), we observed significant CH_4_ accumulation in response to MPn addition well before any increase in C-P lyase transcript abundance (rRNA or protein-coding genes) was observed. No other pathway capable of producing CH_4_ from MPn is known or was recovered in our functional screens. Taken together, these results suggest that even at low relative abundance, the C-P lyase containing organisms can contribute to the CH_4_ supersaturation if MPn is available. In other oligotrophic regions characterized by low P such as the Sargasso or the Mediterranean Seas, where the prevalence of C-P lyase genes in oligotrophic microbes is higher and expression of C-P lyase genes has been reported (Feingersch et al., 2010; Martinez et al., 2010), abundant bacterial groups such as SAR11 could contribute significantly to the CH_4_ supersaturation. In addition, opportunistic C-P lyase-containing microbes identified in this study are abundant in copiotrophic coastal systems, and exhibit transient blooms under specific conditions. For example, several studies have shown that the abundance of *Rhodobacterales* increases after phytoplankton blooms and that expression of their phosphonate ABC transporter genes increases under the resulting P-depleted conditions (Tada et al., 2011; Rinta-Kanto et al., 2012; Teeling et al., 2012). Similarly, Vibrios and Alteromonads, also enriched in the final community of the Glc+N+MPn enrichment and sources of the other two identified C-P lyase clusters, also exhibit increased abundance during diatom blooms (Asplund et al., 2011; Tada et al., 2011; Rinta-Kanto et al., 2012). Finally, the abundance of the MPn-degrading copiotrophs might be higher in the nutrient rich particle-associated fraction than in the free-living fraction. For example, bacterial assemblages in marine aggregates do appear distinct from the surrounding free-living bacterioplankton (DeLong et al., 1993; Crump et al., 1999; Eloe et al., 2010; Smith et al., 2013), and Pn have been shown to be preferentially remineralized relative to phosphate esters in sedimenting particles (Benitez-Nelson et al., 2004).

Ultimately, the contribution of MPn utilization to the CH_4_ levels will depend not only on the P-stress levels and abundance of C-P lyase containing microbes, but on the availability of MPn. Until now MPn has only been shown to be synthesized by the abundant marine archaeon *Nitrosopumilus maritimus*, although genes similar to those involved in MPn biosynthesis in this organism are present in the Global Ocean Survey samples including scaffolds of abundant clades such as SAR11 (Metcalf et al., 2012). Although further analyses of MPn abundance and turnover will be required to evaluate the extent to which MPn degradation contributes to the CH_4_ supersaturation of the oceans, the microbes and processes described here could have a significant impact on our understanding of the CH_4_ cycle in the sea.

## Materials and methods

### Microcosm setup and sampling

Seawater for the microcosm incubation experiments was collected at Station 1 (22° 30.018′ N, 157° 30.033′ W) from 75 m depth at 7:30AM on 7/31/2008 during the Center for Microbial Oceanography: Research and Education (C-MORE) OPEREX cruise. Sampling was conducted using a conductivity-temperature-depth (CTD) rosette sampler aboard the R/V Kilo Moana. CTD and nutrient data were obtained from the HOT program database (http://hahana.soest.hawaii.edu/cmoreDS). Water was transferred to 3 × 20 L acid-washed, sample-water rinsed polycarbonate carboys. Carboys were shaded using black fiberglass screen and placed in blue deckboard incubators to achieve a final light level equivalent to 3% surface irradiance (the *in situ* light intensity at 75 m). The incubator was plumbed with surface seawater to maintain near *in situ* temperatures. The three microcosms were amended as follows: (B1) unamended control; (B2) 100 μM glucose and 16 μM nitrate, hereafter referred to as Glc+N; and (B3) 100 μM glucose, 16 μM nitrate, and 1 μM MPn (Sigma) hereafter referred to as Glc+N+MPn. Microcosm incubations were initiated at ~8:30 AM and subsamples were taken at 0, 3, 6, 12, 24, 36, and 48 h post addition. For DNA extraction, 10 L samples were taken at T0 directly from the Niskin bottle and from each of the three microcosms at 48 h post amendment (TF). Samples were prefiltered though 1.6 μm GF/A filters (47 mm, Whatman), and collected using a 0.22 μm Sterivex filter (Millipore). Three milliliters of lysis buffer (50 mM Tris-HCl, 40 mM EDTA, 0.75 M sucrose, pH 8.3) were added to each filter unit which was then frozen at −20°C. For RNA extraction 1 L subsamples were prefiltered through 1.6 μm GF/A filters (47 mm, Whatman), and microbial biomass was collected onto 0.22 μm Durapore filters (25 mm, Millipore). Filters were immediately transferred to microcentrifuge tubes containing 300 μl RNAlater (Ambion) and frozen at −80°C until extraction.

### CH_4_ measurements

Dissolved CH_4_ concentrations were determined by gas chromatography as previously described (Tilbrook and Karl, 1995). Briefly, water samples were collected in 250 ml glass BOD-type bottles which were filled from the bottom to three times overflowing, aspirated to just below the neck and poisoned with 200 μl saturated mercuric chloride solution. The glass stoppers were replaced after light greasing (Apiezon), secured with polyethylene tape, and stored in the dark at ambient room temperature until analysis. CH_4_ concentrations were determined onboard the ship using purge and trap extraction followed by CH_4_-quantification using a GC-Flame Ionization Detector (Agilent 7890A). Samples were transferred from the BOD bottle to a purge chamber under positive pressure using helium (80 ml/min) and sparged in a 250 mL glass purge chamber for 10 min. The stripped gases were passed through a nafion drier (Perma Pure LLC), and drierite (VWR) before being cold-trapped on a sample loop containing Porapak Q 80/100 (Sigma-Aldrich) and maintained at −80°C using liquid nitrogen. The sample loop was then heated to 90°C and the trapped CH_4_ injected onto a 30 m × 0.32 mm GS-CarbonPLOT analytical capillary column (J&W Scientific) maintained at a temperature of 35°C. The FID was calibrated for CH_4_ using serial dilutions of a 10 ppmv primary standard in nitrogen (Scott-Marin). Data acquisition was performed using ChemStation software version B.03.01.

### Flow cytometry

At each time point, 1 ml of seawater was preserved with glutaraldehyde (0.125% final concentration), frozen in liquid nitrogen, and stored at −80°C for subsequent analysis. Cells were identified after SYBR Green staining (Life Technologies) using an Influx flow cytometer (Becton Dickinson) as previously described (Marie et al., 1997). Data were analyzed using FlowJo software. Cell counts reported are the average of 3 technical replicates.

### Nucleic acid extraction and sequencing

DNA extraction for sequencing and fosmid library construction was performed as previously described (Frias-Lopez et al., 2008). For community RNA extractions, filters were thawed on ice, RNAlater was carefully removed and RNA was extracted using the mirVana RNA isolation kit (Ambion, Austin, TX) as previously described (Shi et al., 2009). Following RNA extraction, gDNA was removed by digestion with Turbo-DNA Free DNAse (Ambion, Austin, TX), and total RNA was concentrated using the RNeasy MinElute Cleanup kit (Qiagen, Valencia, CA).

RNA amplification and cDNA synthesis were performed as described previously (McCarren et al., 2010; Shi et al., 2011). In brief, roughly 10–20 ng of total RNA were amplified using MessageAmp II Bacteria kit (Ambion) and T7*Bpm*IdT_16_VN, an oligo(dT) primer containing a promoter sequence for T7 RNA polymerase and a *Bpm*I recognition site. cDNA was synthesized from the amplified RNA using Superscript III (Invitrogen) and random hexamers for first-strand complementary DNA synthesis, and the SuperScript Double Stranded cDNA Synthesis kit (Invitrogen) for second-strand synthesis. The resulting cDNA was purified using the Qiaquick PCR purification kit (Qiagen), digested with *Bpm*I to remove poly(A) tails and used directly for pyrosequencing. gDNA and cDNA single-stranded libraries were prepared and sequenced using standard GS FLX protocols (454 Life Sciences, Roche) in a Roche Genome Sequencer FLX (Roche Applied Science, Indianapolis, IN).

### Bioinformatic analyses

Reads matching rRNA genes in the gDNA and cDNA databases were identified using BLASTN against a database of 5S, 16S, 18S, 23S, and 28S rRNA sequences derived from the SILVA database (http://www.arb-silva.de) and microbial genomes (Ottesen et al., 2011). Reads aligning with bit scores >50 were parsed out as rRNA sequences and assigned to taxonomic groups according to NCBI taxonomy based on their best match in the SILVA LSU and SSU database. cDNA reads corresponding to known small non coding RNA (sRNA) were identified and mapped to the corresponding sRNA family by BLASTN comparison against the Rfam 10.1 database (http://rfam.sanger.ac.uk) using a bit score cut off of 50.

Non-rRNA, non-sRNA reads were assigned to a single reference gene in the NCBI-nr database (as of September 26, 2011) using BLASTX and a bit score cut-off of 50 as previously described (Ottesen et al., 2011). Reads were assigned to the NCBI taxonomy based on the top hit of the BLASTX comparison against the NCBI-nr database. For this analysis, only reads that could be uniquely assigned to a single taxonomic id were considered. Representation of individual taxa was calculated by normalizing the taxon counts to the number of reads in each library with significant matches in NCBI-nr.

For functional comparison across databases, non-rRNA, non-sRNA sequences were assigned to functional categories based on the KEGG database. The top reference gene(s) in the May 30, 2010 version of the KEGG database matching each read (bit score > 50) was used for annotation. For reads matching multiple reference genes with equal bit score, all top hits were retained with its representation scaled proportionally to the number of top hits (Stewart et al., 2011). Ortholog counts for each sample were normalized to the total number of non-rRNA, non-sRNA sequences with significant matches to the KEGG database. KEGG ortholog (KO) abundances were compared across libraries using the AC Test (Audic and Claverie, 1997) and a false discovery rate correction for multiple comparisons (Benjamini and Hochberg, 1995) as previously described (Ottesen et al., 2011). Only KO with FDR <0.05 were considered significant in this analysis.

KEGG pathway counts were generated based on the total number of sequences assigned to KO annotations within that pathway. As described above for KO, pathway representation was calculated normalizing pathway counts to the total number of non-rRNA, non-sRNA sequences with significant matches to the KEGG database. Because some of the most abundantly expressed orthologs in the amendments are involved in processes not included in the standard KEGG pathways we modified the KEGG pathway database to better capture the observed changes by annotating three relevant and well-characterized microbial pathways: alkylphosphonate metabolism, phosphate metabolism, and polyhydroxyalkanoate biosynthesis. Only KEGG orthologs with assigned KO numbers and clear genetic or biochemical evidence to support their assignment were included in each of these pathways (see list in Table S4). Read counts across KEGG pathways were used to cluster non-rRNA, non-sRNA cDNA datasets based on shared gene content using GenePattern software (http://genepattern.broadinstitute.org) (Reich et al., 2006). Samples were clustered using hierarchical clustering based on pairwise average-linkage method with Pearson correlation coefficients as the distance measure. Only pathways representing >1% of total KO reads in at least one time point were included in the cluster analysis.

Abundance of Pn-degrading microbes in our metagenomic libraries was assessed as previously described (Martinez et al., 2010). Briefly, predicted protein sequences of known phn genes and *E. coli* RecA and GyrB as single copy reference were used as query to interrogate available databases using NCBI TBLASTN and a bit score cut off value of 50. Gene counts were size normalized using the query protein length. The percentage of microbes containing each gene was calculated assuming that *recA* is present in single copy in every microbial genome (Howard et al., 2008; Reisch et al., 2008). For each identified C-P lyase gene, putative taxonomic affiliation was assigned based on its top scoring hit in the NCBI-nr databse.

The expression of predicted proteins in the fosmids identified in MPn utilization functional screens was determined as follows. Predicted protein sequences were used to query the 48 h metagenomic and metatranscriptomic databases from the Glc+N+MPn enrichment (B3) using TBLASTN. The number of reads matching each predicted protein (bit score >50, % identity >95) was normalized to the total number of reads in the library. Expression ratio was expressed as the ratio (non-rRNA non-sRNA reads per gene/total non-rRNA non-sRNA reads)/(gDNA reads matching gene/total gDNA reads).

### Functional screening

A large-insert metagenomic DNA library was constructed from the Glc+N+MPn final DNA sample (B3_TF) using fosmid vector pCC1FOS (Epicentre) as previously described (DeLong et al., 2006). The resulting phage lysate was used to infect *E. coli* EPI300. Approximately 47,000 fosmid-containing colonies (~1.4 Gb metagenomic DNA) were pooled and used to prepare fosmid DNA by alkaline lysis followed by cesium chloride ultracentrifugation (Sambrook et al., 1989). The resulting B3_TF metagenomic library was screened for MPn utilization as previously described (Martinez et al., 2010). MPn positive clones were de-replicated by restriction analyses and sequenced using transposon mutagenesis as described (Martinez et al., 2007). The complete DNA sequence was assembled using Sequencher v. 4.10 (Gene Codes Corporation, Ann Arbor, MI) and annotated with FGENESB (Softberry) and BlastP (NCBI). Pn specificity was evaluated in microtiter plate liquid cultures (MOPS glycerol medium with 0.1 mM of the indicated P source) as described in (Martinez et al., 2010) except that culture absorbance (600 nm) was used to measure growth.

### GenBank accession numbers

Fosmid sequences have been submitted to GenBank and are available under accession numbers KF742554-KF742556. The 454 metagenomic and metatranscriptomic data had been deposited with the Short Reads Archive (NCBI) under Accession No. SRP030766.

### Conflict of interest statement

The authors declare that the research was conducted in the absence of any commercial or financial relationships that could be construed as a potential conflict of interest.
