# Supplementary material for: Metatranscriptomic and functional metagenomic analysis of methylphosphonate utilization by marine bacteria
Source: Front Microbiol. 2013 Nov 26;4:340. doi: 10.3389/fmicb.2013.00340 (PMC3840354; doi:10.3389/fmicb.2013.00340)
Supplement: Supplementary file 1 [file DataSheet1.PDF]

SUPLEMENTARY ONLINE MATERIAL

Metatranscriptomics of methylphosphonate utilization by marine bacteria

SUPPLEMENTARY FIGURE LEGENDS

Figure S1. Vertical distributions of physical and chemical water properties at the time of sampling. Mesocosms were set up with 75 m depth water (red line), directly above deep chlorophyll maximum. Concentration of phosphorus at 75m was 50 nmol/kg. Data were obtained from Hawaii Ocean Time Series (http://hahana.soest.hawaii.edu/cmoreDS/cdisplay.html).

Figure S2. Experimental set up. Three microcosms containing 20L of sea water were amended as follows: B1: no amendment control; B2: amended with 100 μM glucose and 16 μM nitrate; B3: amended with 100 μM glucose,16 μM nitrate, and 1μM MPn. Microcosms were incubated for 48 h and subsampled at the indicated times for community RNA and methane measurements. Community DNA was sampled before amendment and at the end of the 48 h incubation. See Material and Methods for details.

Figure S3. Microbial population composition assessed by taxonomic classification of LSU and SSU rRNA reads at the order level. Left panel, genomic DNA datasets. Right panel, cDNA reads at different time points after addition of Glc+N+MPn. Only taxonomic groups that represent > 2% of total assigned reads in at least one dataset have been included with all other groups binned together with unassigned reads. Abundance expressed as the percentage of reads assigned to each taxon divided by the total number of assigned reads in each database.

Figure S4. The twenty most abundant taxa in selected datasets based on the NCBI taxonomic affiliation of protein-coding reads matching metatranscriptomic and metagenomic reads. For this analysis, only those reads that could be uniquely assigned to a single taxon id were considered. Abundance expressed as the percentage of reads assigned to each taxon divided by the total number of assigned reads in each database. Average % amino acid identity across all reads assigned to each taxon is indicated in parenthesis next to the taxon name.

Figure S5. Expression ratio of fosmid genes 48 h after addition of Glc+N+MPn. Predicted proteins where used to recruit RNA and gDNA sequences (cut off b > 50, id >95%). Expression ratio = (% of matching reads in cDNA)/(% of matching reads in gDNA). Categories in the x-axis represent predicted genes. The position of the C-P lyase cluster in each fosmid is indicated.

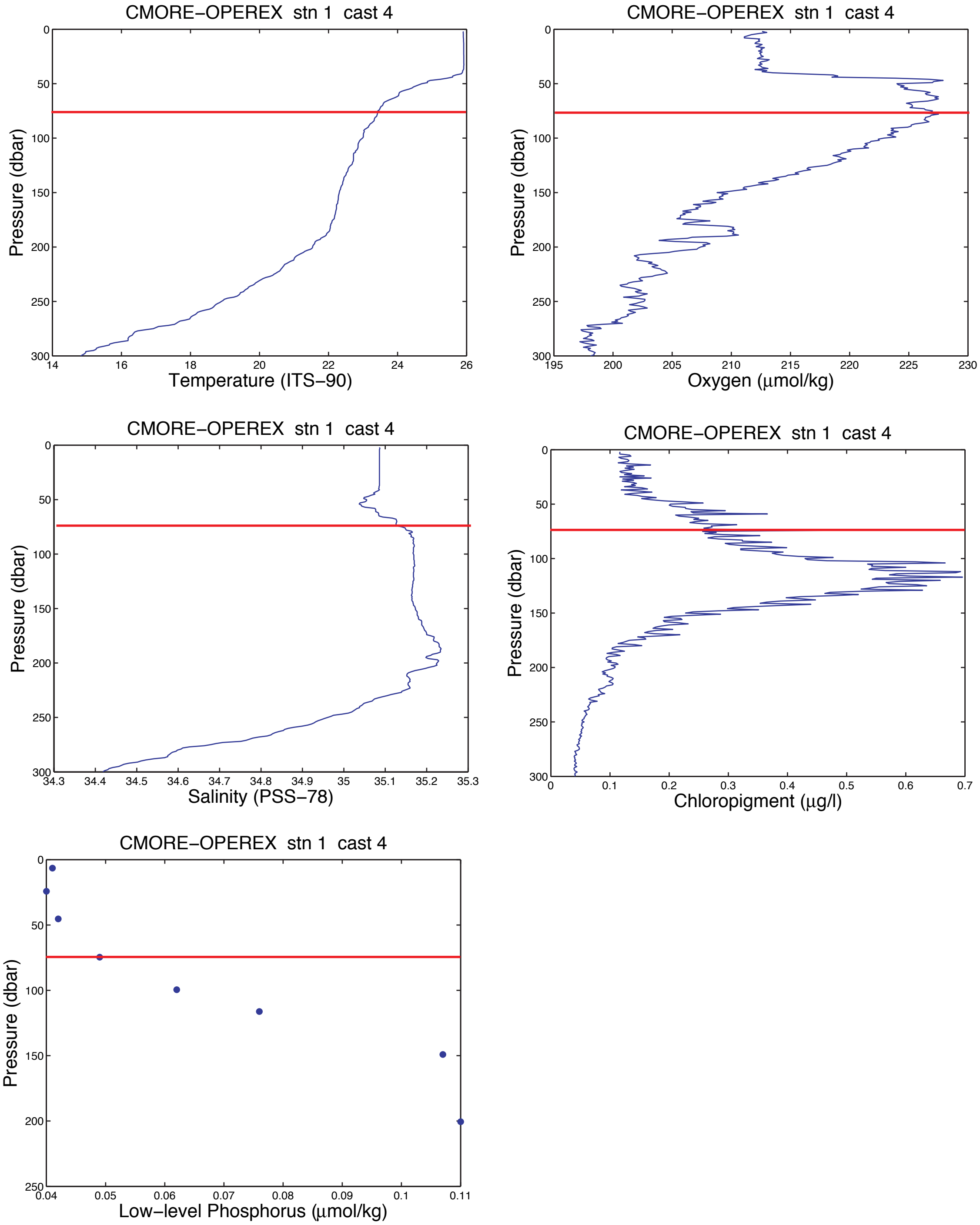

Figure S1. Vertical profiles of seawater at the time of sampling. Mesocosms were set up with 75 m depth water (red line), directly above deep chlorophyll maximum. Concentration of phosphorus at 75m was 50 nmol/kg. Data were obtained from Hawaii Ocean Time Series (http://hahana.soest.hawaii.edu/cmoreDS/cdisplay.html).

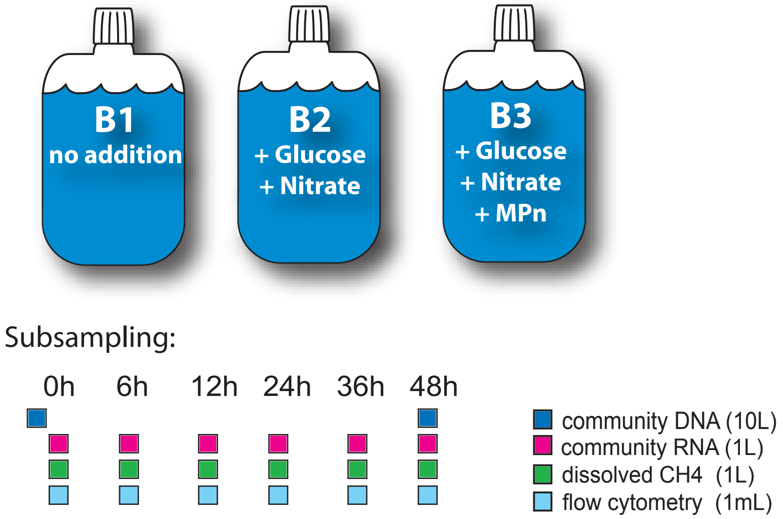

Figure S2. Experimental set up. Three microcosms containing 20L of sea water were amended as follows: B1: no amendment control; B2: amended with 100 µM glucose and 16 µM nitrate; B3: amended with 100 µM glucose,16 µM nitrate, and 1µM MPn. Microcosms were incubated for 48 h and subsampled at the indicated times for community RNA and methane measurements. Community DNA was sampled before amendment and at the end of the 48 h incubation. See Material and Methods for details.

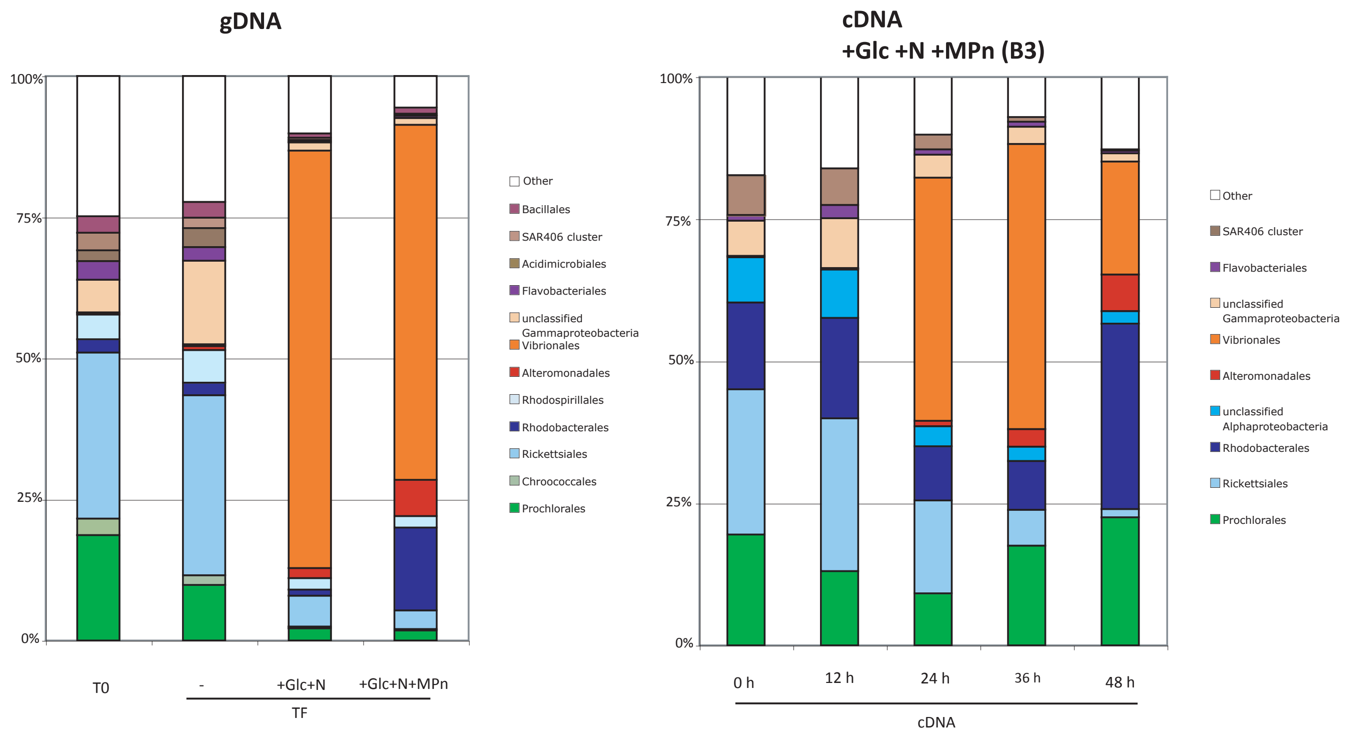

Figure S3. Microbial population composition assessed by taxonomic classification of LSU and SSU rRNA reads at the order level. Left panel, genomic DNA datasets. Right panel, cDNA reads at different time points after addition of Glc+N+MPn. Only taxonomic groups that represent > 2% of total assigned reads in at least one dataset have been included with all other groups binned together with unassigned reads. Abundance expressed as the percentage of reads assigned to each taxon divided by the total number of assigned reads in each database.

Figure S4

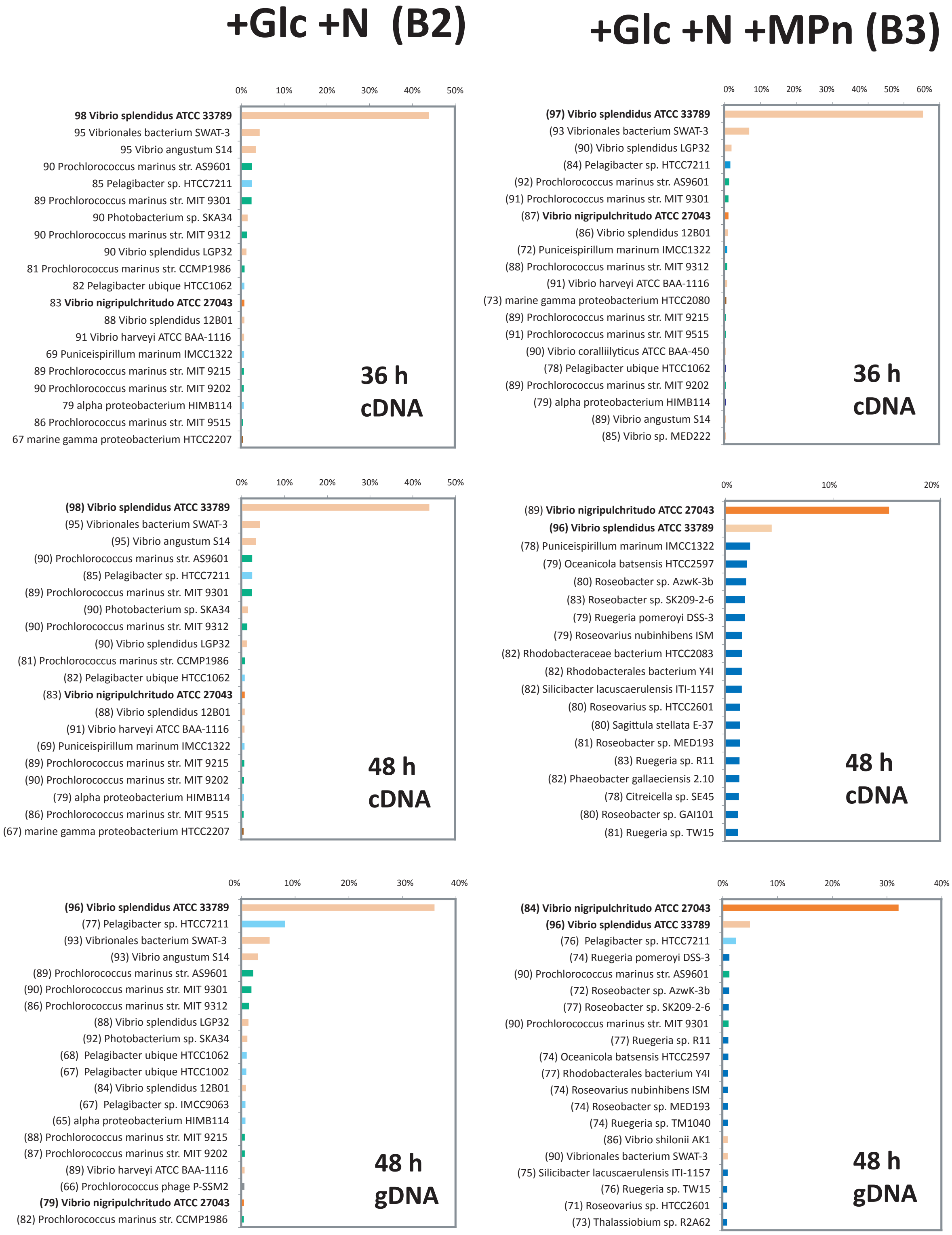

Figure S4. The twenty most abundant taxa in selected datasets based on the NCBI taxonomic affiliation of protein-coding reads matching metatranscriptomic and metagenomic reads. For this analysis, only those reads that could be uniquely assigned to a single taxon id were considered. Abundance expressed as the percentage of reads assigned to each taxon divided by the total number of assigned reads in each database. Average % amino acid identity across all reads assigned to each taxon is indicated in parenthesis next to the taxon name.

Figure S5

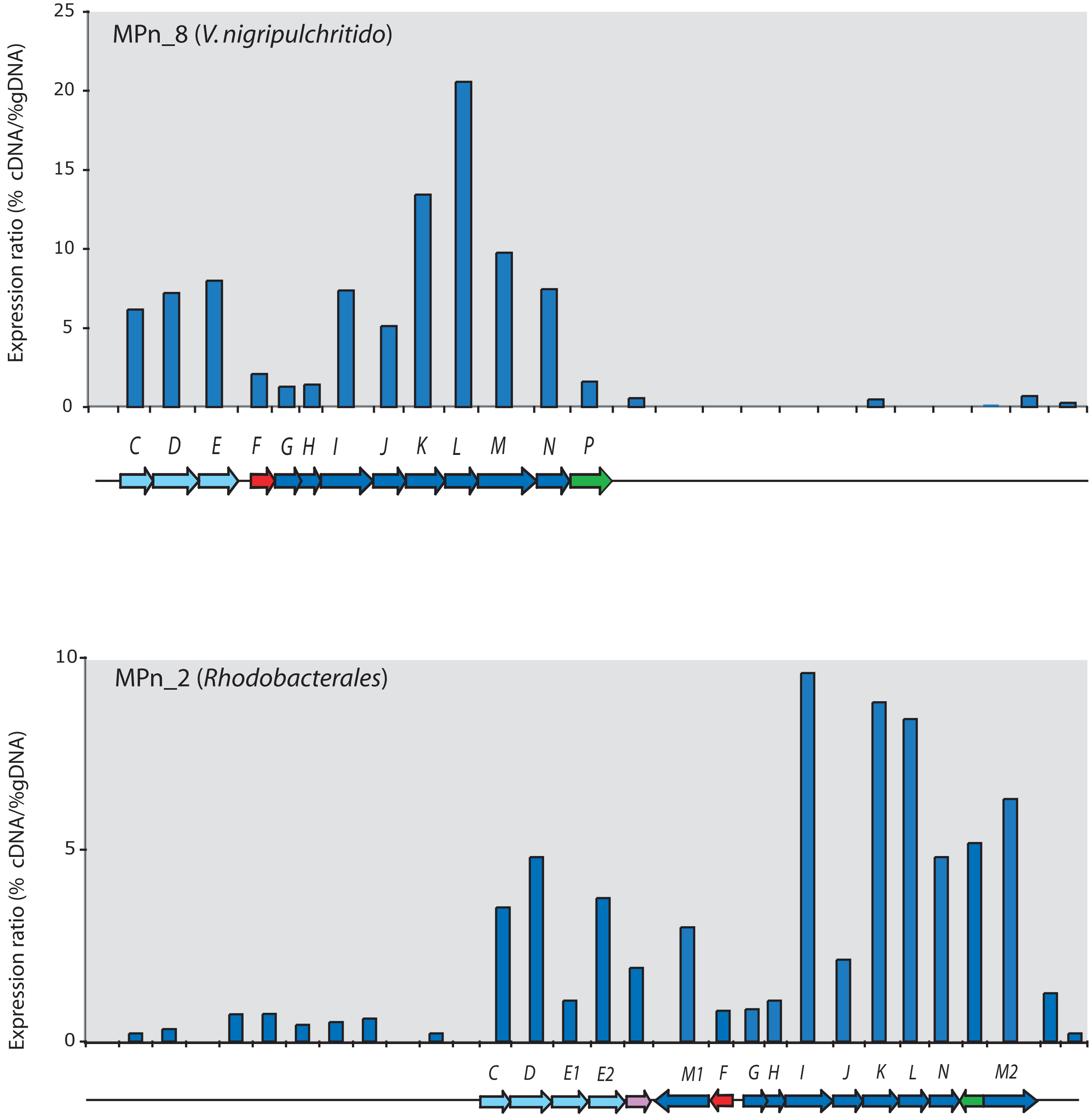

Figure S5. Expression ratio of fosmid genes 48 h after addition of Glc+N+MPn. Predicted proteins where used to recruit RNA and gDNA sequences (cut off b > 50, id >95%). Expression ratio = (% of matching reads in cDNA)/(% of matching reads in gDNA). Categories in the x-axis represent predicted genes. The position of the C-P lyase cluster in each fosmid is indicated.

Table S1. Taxonomic affiliation of C-P lyase hits in B3 (Glc+N+MPn) 48 h gDNA sample

Order	Organism	C-P lyase hit counts	Sum	% of all CP lyase hits
Vibrionales	*Vibrio nigripulchritudo ATCC 27043*	73	74	46.0%
	Vibrio shilonii AK1	1		
Rhodobacterales	*Oceanibulbus indolifex HEL-45*	9	83	51.6%
	Ruegeria sp. R11	8		
	Roseobacter sp. AzwK-3b	7		
	Oceanicola batsensis HTCC2597	6		
	Roseovarius nubinhibens ISM	5		
	Roseovarius sp. TM1035	5		
	Roseobacter litoralis Och 149	4		
	Jannaschia sp. CCS1	3		
	Octadecabacter antarcticus 238	3		
	Rhodobacterales bacterium HTCC2654	3		
	Rhodobacterales bacterium Y4I	3		
	Roseobacter sp. GAI101	3		
	Roseobacter sp. MED193	3		
	Roseovarius sp. 217	3		
	Roseovarius sp. HTCC2601	3		
	Ruegeria pomeroyi DSS-3	3		
	Sulfitobacter sp. NAS-14.1	3		
	Octadecabacter antarcticus 307	2		
	Paracoccus denitrificans PD1222	2		
	Paracoccus sp. TRP	2		
	Silicibacter sp. TrichCH4B	2		
	Roseobacter sp. SK209-2-6	1		
Rhizobiales	*Mesorhizobium opportunistum WSM2075*	2	2	1.2%
Clostridiales	*Desulfotomaculum kuznetsovii DSM 6115*	1	1	0.6%
Chloroflexales	*Oscillochloris trichoides DG6*	1	1	0.6%

Table S2. Fraction of KEGG ortholog groups (k0) whose abundance changes significantly in metatranscritome pairwise comparisons. AC-tests (FDR <0.05)

Time (h)	B2 *vs* B1<br>Glc+N *vs* Ctl	B3 ctl *vs* B2<br>Glc+N+MPn *vs* Ctl	B3 vs B2<br>Glc+N+MPn *vs* Glc+N
0	4.7%	2.0%	2.5%
12	2.6%	3.2%	0.5%
24	17.9%	17.3%	4.0%
36	12.9%	15.5%	10.9%
48	25.6%	12.7%	11.9%

Table S3. Ten most abundant transcripts in each metatranscriptome.

T0h

No amendment control (B1)

Kegg_ko	Description	Abundance[1]
K01601	rbcL; ribulose bisophosphate carboxylase large chain	5.8
K04077	groEL; chaperonin GroEL	4.3
K02358	tuf; elongation factor Tu	3.5
K02690	psaB; photosystem I P700 chlorophyll a apoprotein A2	3.1
K03798	ftsH; cell division protease	1.7
K02689	psaA; photosystem I P700 chlorophyll a apoprotein A1	1.4
K03046	rpoC; DNA-directed RNA polymerase beta' subunit	1.4
K00962	pnp; polynucleotide phosphorylase/polyadenylase	1.4
K02699	psaL; photosystem I reaction center protein subunit XI	1.2
K04043	chaperone protein DnaK	1.1

Glc+N amendment (B2)

Kegg_ko	Description	Abundance
K01601	rbcL; ribulose bisophosphate carboxylase large chain	12.1
K02358	tuf; elongation factor Tu	6.7
K04077	groEL; chaperonin GroEL	4.5
K02690	psaB; photosystem I P700 chlorophyll a apoprotein A2	3.6
K08918	pcbA; chlorophyll a/b binding light-harvesting complex protein PcbA	3.0
K02706	psbD; photosystem II D2 protein	2.1
K03798	ftsH; cell division protease	1.8
K02704	psbB; photosystem II P680 chlorophyll A apoprotein	1.6
K02699	psaL; photosystem I reaction center protein subunit XI	1.5
K00962	pnp; polynucleotide phosphorylase/polyadenylase	1.1

Glc+N+MPn amendment (B3)

Kegg_ko	Description	Abundance
K01601	rbcL; ribulose bisophosphate carboxylase large chain	7.2
K04077	groEL; chaperonin GroEL	6.6
K02358	tuf; elongation factor Tu	5.6
K02690	psaB; photosystem I P700 chlorophyll a apoprotein A2	3.2
K03798	ftsH; cell division protease	1.9
K08918	pcbA; chlorophyll a/b binding light-harvesting complex protein PcbA	1.8
K03046	rpoC; DNA-directed RNA polymerase beta' subunit	1.5
K00962	pnp; polynucleotide phosphorylase/polyadenylase	1.4
K02699	psaL; photosystem I reaction center protein subunit XI	1.4
K02706	psbD; photosystem II D2 protein	1.4

T12h

No amendment control (B1)

Kegg_ko	Description	Abundance
K02358	tuf; elongation factor Tu	2.6
K04077	groEL; chaperonin GroEL	1.7
K02690	psaB; photosystem I P700 chlorophyll a apoprotein A2	1.6
K03043	rpoB; DNA-directed RNA polymerase subunit beta	1.4
K02274	cytochrome c oxidase subunit I	1.2
K03046	rpoC; DNA-directed RNA polymerase beta' subunit	1.2
K03798	ftsH; cell division protease	1.2
K01601	rbcL; ribulose bisophosphate carboxylase large chain	1.1
K02112	F0F1 ATP synthase subunit beta	0.9
K00962	pnp; polynucleotide phosphorylase/polyadenylase	0.9

Glc+N amendment (B2)

Kegg_ko	Description	Abundance
K02358	tuf; elongation factor Tu	5.1
K04077	groEL; chaperonin GroEL	2.8
K03043	rpoB; DNA-directed RNA polymerase subunit beta	1.6
K01738	cysteine synthase A	1.5
K01999	branched-chain amino acid ABC transporter, substrate-binding protein	1.5
K03046	rpoC; DNA-directed RNA polymerase beta' subunit	1.5
K02690	psaB; photosystem I P700 chlorophyll a apoprotein A2	1.4
K02274	cytochrome c oxidase subunit I	1.3
K02055	ABC transporter, spermidine/putrescine substrate-binding protein	1.3
K03798	ftsH; cell division protease	1.2

Glc+N+MPn amendment (B3)

Kegg_ko	Description	Abundance
K02358	tuf; elongation factor Tu	5.0
K04077	groEL; chaperonin GroEL	3.1
K01738	cysteine synthase A	1.8
K02690	psaB; photosystem I P700 chlorophyll a apoprotein A2	1.7
K01601	rbcL; ribulose bisophosphate carboxylase large chain	1.5
K03046	rpoC; DNA-directed RNA polymerase beta' subunit	1.5
K03043	rpoB; DNA-directed RNA polymerase subunit beta	1.4
K03798	ftsH; cell division protein FtsH	1.4
K01999	branched-chain amino acid ABC transporter, periplasmic substrate-bindir	1.3
K08918	pcbA; light-harvesting complex protein	1.3

T24h

No amendment control (B1)

Kegg_ko	Description	Abundance
K01601	rbcL; ribulose bisophosphate carboxylase large chain	4.4
K03798	ftsH; cell division protease	3.7
K02358	tuf; elongation factor Tu	3.6
K04077	groEL; chaperonin GroEL	3.6
K02703	psbA; photosystem II PsbA protein D1	2.7
K02706	psbD; photosystem II PsbD protein	2.1
K01738	cysteine synthase A	1.6
K02705	psbC; photosystem II PsbC protein	1.3
K06890	hypothetical protein; K06890	1.2
K03046	rpoC; DNA-directed RNA polymerase beta' subunit	1.1

Glc+N amendment (B2)

Kegg_ko	Description	Abundance
K02040	ptsS; phosphate ABC transporter, periplasmic phosphate-binding protein	4.6
K01601	rbcL; ribulose bisophosphate carboxylase large chain	4.6
K02777	PTS system, glucose-specific IIA component	4.5
K00626	phbA, atoB; acetyl-CoA acetyltransferase	4.3
K04077	groEL; chaperonin GroEL	2.9
K02358	tuf; elongation factor Tu	2.8
K06194	lipoprotein NlpD cell division protein	1.9
K03798	ftsH; cell division protease	1.6
K01738	cysteine synthase A	1.6
K00023	phbB; acetoacetyl-CoA reductase	1.5

Glc+N+MPn amendment (B3)

Kegg_ko	Description	Abundance
K02040	ptsS; phosphate ABC transporter, periplasmic phosphate-binding protein	4.6
K02777	PTS system, glucose-specific IIA component	3.5
K04077	groEL; chaperonin GroEL	2.8
K01601	rbcL; ribulose bisophosphate carboxylase large chain	2.7
K06194	lipoprotein NlpD cell division protein	2.3
K00626	phbA, atoB; acetyl-CoA acetyltransferase	2.2
K02358	tuf; elongation factor Tu	2.2
K03798	ftsH; cell division protease	2.1
K01738	cysteine synthase A	1.8
K02706	psbD; photosystem II D2 protein	1.2

T36h

No amendment control (B1)

Kegg_ko	Description	Abundance
K02358	tuf; elongation factor Tu	3.8
K02690	psaB; photosystem I P700 chlorophyll a apoprotein A2	2.7
K04077	groEL; chaperonin GroEL	1.7
K08918	pcbA; chlorophyll a/b binding light-harvesting complex protein PcbA	1.7
K03046	rpoC; DNA-directed RNA polymerase beta' subunit	1.5
K01999	branched-chain amino acid ABC transporter, substrate-binding protein	1.3
K02274	cytochrome c oxidase subunit I	1.3
K03043	rpoB; DNA-directed RNA polymerase subunit beta	1.3
K03798	ftsH; cell division protease	1.1
K01738	cysteine synthase A	1.1

Glc+N amendment (B2)

Kegg_ko	Description	Abundance
K02040	ptsS; phosphate ABC transporter, periplasmic phosphate-binding protein	5.8
K06194	lipoprotein NlpD cell division protein	2.5
K00626	phbA, atoB; acetyl-CoA acetyltransferase	2.3
K02358	tuf; elongation factor Tu	1.7
K07689	uvrY; two-component system invasion response regulator UvrY	1.3
K00023	phbB; acetoacetyl-CoA reductase	1.3
K02699	psaL; photosystem I reaction center protein subunit XI	1.1
K01077	phoA; alkaline phosphatase	1.1
K02777	PTS system, glucose-specific IIA component	1.0
K01868	thrS; threonyl-tRNA synthetase	1.0

Glc+N+MPn amendment (B3)

Kegg_ko	Description	Abundance
K02040	ptsS; phosphate ABC transporter, periplasmic phosphate-binding protein	3.4
K00626	phbA, atoB; acetyl-CoA acetyltransferase	2.0
K06194	lipoprotein NlpD cell division protein	1.2
K02358	tuf; elongation factor Tu	1.0
K00023	phbB; acetoacetyl-CoA reductase	1.0
K02037	pstC; phosphate ABC transporter, permease protein	1.0
K02355	fusA; elongation factor G	0.8
K03406	mcp; methyl-accepting chemotaxis protein	0.8
K01868	thrS; threonyl-tRNA synthetase	0.7
K02406	flaE; flagellin; K02406 flagellin	0.7

T48h

No amendment control (B1)

Kegg_ko	Description	Abundance
K03798	ftsH; cell division protease	1.8
K02703	psbA; photosystem II PsbA protein D1	1.4
K02706	psbD; photosystem II PsbD protein	1.3
K02355	fusA; elongation factor G	1.0
K02358	tuf; elongation factor Tu	1.0
K02274	cytochrome c oxidase subunit I	0.9
K06890	hypothetical protein; K06890	0.8
K04077	groEL; chaperonin GroEL	0.8
K04043	chaperone protein DnaK	0.8
K02707	psbE; photosystem II cytochrome b559 subunit alpha	0.8

Glc+N amendment (B2)

Kegg_ko	Description	Abundance
K02040	ptsS; phosphate ABC transporter, periplasmic phosphate-binding protein	1.4
K07093	phoX; phosphatase	1.2
K03406	mcp; methyl-accepting chemotaxis protein	0.8
K02358	tuf; elongation factor Tu	0.8
K02055	ABC transporter, spermidine/putrescine substrate-binding protein	0.8
K02703	psbA; photosystem II PsbA protein D1	0.7
K02406	flaE; flagellin; K02406 flagellin	0.7
K02355	fusA; elongation factor G	0.7
K01533	copA; Cu2+-exporting ATPase	0.6
K03798	ftsH; cell division protease	0.5

Glc+N+MPn amendment (B3)

Kegg_ko	Description	Abundance
K03043	rpoB; DNA-directed RNA polymerase subunit beta	0.8
K00265	gltB; glutamate synthase subunit alpha	0.7
K03046	rpoC; DNA-directed RNA polymerase beta' subunit	0.7
K02027	ABC multiple sugar transporter substrate-binding protein	0.7
K00372	nitrate reductase catalytic subunit	0.7
K02355	fusA; elongation factor G	0.6
K03089	rpoH; RNA polymerase factor sigma-32	0.6
K02358	tuf; elongation factor Tu	0.6
K06162	phnM; phosphonate metabolism protein PhnM	0.5
K04077	groEL; chaperonin GroEL	0.5

Top 10 genes identified via BlastX comparisson to KEGG ortholog database. Significance cutoff, bits>50.

[1] Abundance expressed as percent of total reads mapped to KEGG orthologs with b>50.

Table S4. Microbial metabolic pathways added to KEGG pathway database

**Polyhydroxyalkanoate metabolism**	
K05973	phaZ; poly(3-hydroxybutyrate) depolymerase [EC:3.1.1.75]
K00023	phbB; acetoacetyl-CoA reductase [EC:1.1.1.36]
K00626	phbA, atoB; acetyl-CoA C-acetyltransferase [EC:2.3.1.9]
K03821	phbC, phaC; polyhydroxyalkanoate synthase [EC:2.3.1.-]
**Alkylphosphonate metabolism**	
K02044	phnD; phosphonate transport system substrate-binding protein
K02042	phnE; phosphonate transport system permease protein
K02041	phnC; phosphonate transport system ATP-binding protein
K02043	phnF; phosphonate transport system regulatory protein
K06166	phnG; phosphonate metabolism PhnG protein
K06165	phnH; carbon-phosphorus lyase complex subunit PhnH protein
K06164	phnI; phosphonate metabolism protein
K06163	phnJ; C-P (carbon-phosphorus) lyase component protein
K05781	phnK; putative phosphonate transport system ATP-binding protein
K05780	phnL; putative phosphonate transport system ATP-binding protein
K06162	phnM; phosphonate metabolism protein PhnM
K05774	phnN ; ribose 1,5-bisphosphokinase [EC:2.7.4.23]
K09994	phnO;aminoalkylphosphonic acid N-acetyltransferase
K06167	phnP; carbon-phosphorus lyase complex accessory protein
**Phosphate metabolism**	
K02040	pstS; phosphate transport system substrate-binding protein
K02037	pstC; phosphate transport system permease protein
K02038	pstA; phosphate transport system permease protein
K02036	pstB; phosphate transport system ATP-binding protein [EC:3.6.3.27]
K00858	ppnK; inorganic polyphosphate/ATP-NAD kinase (EC:2.7.1.23)
K00937	ppk; polyphosphate kinase (EC:2.7.4.1)
K00947	polyphosphate:AMP phosphotransferase (EC:2.7.4.-)
K01077	phoA; alkaline phosphatase [EC:3.1.3.1]
K07093	phoX; putative monomeric alkaline phosphatase
K01113	phoD; alkaline phosphatase D [EC:3.1.3.1]
K02039	phoU; phosphate uptake regulator, PhoU
K07657	phoB; two-component system phosphate regulon response regulator
K07658	phoP; two-component system phosphate regulon response regulator PhoP
K07636	phoR; two-component system phosphate regulon sensor histidine kinase PhoR

Table S5. KEGG Pathway abundance in metatranscriptomomic datasets sorted by maximum fold change vs conttrol
(only pathways representing >1% of total assigned reads in at least one dataset are included)

		ABUNDANCE (% of total assigned reads)															FOLD CHANGE (% treatment / % control)														
		Control (B1)					+Glc+N (B2)					+Glc+N+MPn (B3)					+Glc+N/control (B2/B1)					+Glc+N+MPn/control (B3/B1)					+Glc+N+MPn/+Glc+N (B3/B2)				
Annotation		0 h	12 h	24 h	36 h	48 h	0 h	12 h	24 h	36 h	48 h	0 h	12 h	24 h	36 h	48 h	0 h	12 h	24 h	36 h	48 h	0 h	12 h	24 h	36 h	48 h	0 h	12 h	24 h	36 h	48 h
**Phosphotransferase system (PTS)**	PATH:ko02060	0.1	0.0	0.0	0.1	0.1	0.1	0.0	4.1	1.1	0.3	0.1	0.0	3.1	0.5	0.3	1	5	92	10	5	1	5	69	5	5	1	1	1	0	1
**Alkylphosphonate metabolism**	PATH:ko00441	0.0	0.1	0.0	0.0	0.0	0.0	0.0	0.0	0.0	0.0	0.0	0.0	0.0	0.0	2.0	0	1	1	1	2	0	0	1	1	80	1	0	1	1	40
**Phosphate metabolism**	PATH:ko00442	0.3	0.3	0.2	0.1	0.2	0.1	0.1	4.6	6.5	3.1	0.2	0.1	4.4	5.0	1.4	0	0	21	44	13	1	0	20	34	6	3	1	1	1	0
**Polyhydroxyalkanoate metabolism**	PATH:ko00505	0.1	0.2	0.2	0.2	0.4	0.2	0.1	5.0	3.1	0.7	0.1	0.3	2.9	2.4	0.5	1	1	28	16	2	1	2	16	13	1	1	2	1	1	1
**Synthesis and degradation of ketone bodies**	PATH:ko00072	0.1	0.2	0.2	0.2	0.4	0.2	0.1	3.5	1.8	0.3	0.1	0.3	1.7	1.6	0.3	1	1	19	11	1	1	2	9	9	1	1	3	0	1	1
**Two-component system**	BR:ko02022	0.4	0.3	0.3	0.1	0.5	0.1	0.2	1.4	2.0	1.4	0.2	0.2	1.5	1.6	1.0	0	1	4	15	3	1	1	4	12	2	2	1	1	1	1
**Starch and sucrose metabolism**	PATH:ko00500	0.4	0.6	0.3	0.3	0.5	0.3	0.3	4.2	1.6	1.0	0.3	0.4	3.3	1.2	0.7	1	0	14	5	2	1	1	11	3	1	1	2	1	1	1
**Bacterial chemotaxis**	PATH:ko02030	0.1	0.2	0.2	0.2	0.2	0.1	0.1	0.9	1.2	1.4	0.1	0.1	0.8	1.5	0.7	2	1	5	7	6	2	0	5	9	3	1	0	1	1	0
**Amino sugar and nucleotide sugar metabolism**	PATH:ko00520	0.7	0.7	0.5	0.6	1.0	0.5	0.4	4.5	1.7	1.1	0.6	0.6	3.5	1.2	0.9	1	1	8	3	1	1	1	6	2	1	1	1	1	1	1
**Vibrio cholerae pathogenic cycle**	PATH:ko05111	0.2	0.2	0.1	0.2	0.2	0.2	0.2	0.6	0.8	0.8	0.2	0.2	0.6	1.2	0.3	1	1	6	5	3	1	1	5	7	1	1	1	1	1	0
**Two-component system**	PATH:ko02020	1.4	1.8	1.7	1.7	2.0	1.6	1.7	10.1	10.6	4.9	1.5	1.8	8.5	8.0	2.9	1	1	6	6	2	1	1	5	5	1	1	1	1	1	1
**Folate biosynthesis**	PATH:ko00790	0.7	0.2	0.4	0.2	0.4	0.3	0.1	0.4	1.0	0.6	0.4	0.2	0.4	0.7	0.4	0	1	1	6	1	1	1	1	4	1	1	1	1	1	1
**Benzoate degradation via CoA ligation**	PATH:ko00632	0.5	0.8	0.7	0.9	1.2	0.6	0.9	3.9	2.2	1.0	0.6	0.9	2.1	2.1	1.1	1	1	6	2	1	1	1	3	2	1	1	1	1	1	1
**Terpenoid backbone biosynthesis**	PATH:ko00900	0.8	0.7	0.7	0.5	1.0	0.6	0.5	3.7	2.1	0.8	0.7	0.6	2.1	1.9	0.7	1	1	5	4	1	1	1	3	4	1	1	1	1	1	1
**Bacterial motility proteins**	BR:ko02035	0.5	0.8	0.5	0.7	1.3	0.7	0.6	1.5	2.3	3.4	0.5	0.5	1.6	3.5	1.7	1	1	3	3	3	1	1	3	5	1	1	1	1	1	0
**Transcription factors**	BR:ko03000	0.3	0.3	0.2	0.3	0.7	0.2	0.3	0.6	1.0	1.6	0.2	0.3	0.7	1.7	1.1	1	1	3	3	2	0	1	3	5	2	1	1	1	2	1
**Lysine degradation**	PATH:ko00310	0.7	0.8	1.0	1.1	1.2	0.8	1.2	3.9	2.3	0.7	0.7	1.1	2.2	1.9	1.1	1	2	4	2	1	1	1	2	2	1	1	1	1	1	2
**Flagellar assembly**	PATH:ko02040	0.4	0.7	0.4	0.5	1.1	0.6	0.5	0.8	1.3	1.9	0.4	0.4	0.9	2.0	0.9	1	1	2	2	2	1	1	2	4	1	1	1	1	2	0
**Butanoate metabolism**	PATH:ko00650	1.6	1.6	1.7	1.9	2.0	1.6	2.1	6.0	4.2	1.8	1.8	2.2	3.9	3.5	2.0	1	1	3	2	1	1	1	2	2	1	1	1	1	1	1
**Fatty acid metabolism**	PATH:ko00071	0.9	1.0	1.2	1.5	1.6	1.1	1.6	4.2	2.4	0.9	0.9	1.5	2.4	2.1	1.3	1	2	3	2	1	1	1	2	1	1	1	1	1	1	1
**Glycolysis / Gluconeogenesis**	PATH:ko00010	2.1	2.1	1.8	2.0	1.8	2.1	2.3	5.9	3.2	1.8	2.4	2.4	4.9	2.2	1.9	1	1	3	2	1	1	1	3	1	1	1	1	1	1	1
**Tryptophan metabolism**	PATH:ko00380	0.8	1.1	1.3	1.6	1.3	1.0	1.6	4.1	2.4	0.8	0.9	1.5	2.3	2.0	1.2	1	2	3	1	1	1	1	2	1	1	1	1	1	1	1
**Valine, leucine and isoleucine degradation**	PATH:ko00280	1.0	1.3	1.5	1.6	1.8	1.2	1.7	4.2	2.4	0.8	1.1	1.8	2.4	2.0	1.4	1	1	3	1	0	1	1	2	1	1	1	1	1	1	2
**Bacterial secretion system**	PATH:ko03070	0.9	0.8	0.7	0.4	0.9	0.5	0.6	1.5	1.1	1.0	0.6	0.6	1.4	1.0	1.1	1	1	2	3	1	1	1	2	3	1	1	1	1	1	1
**Propanoate metabolism**	PATH:ko00640	1.2	1.7	1.7	1.9	1.9	1.5	2.3	4.6	2.7	1.1	1.7	2.2	2.7	2.3	1.5	1	1	3	1	1	1	1	2	1	1	1	1	1	1	1
**ABC transporters**	PATH:ko02010	2.2	3.1	3.3	3.6	1.8	2.9	4.4	7.2	8.1	4.0	2.3	4.1	6.6	7.0	5.1	1	1	2	2	2	1	1	2	2	3	1	1	1	1	1
**Protein export**	PATH:ko03060	0.9	0.8	0.6	0.4	0.8	0.5	0.6	1.4	1.0	0.7	0.7	0.6	1.3	0.9	1.0	1	1	2	3	1	1	1	2	2	1	1	1	1	1	1
**Pyruvate metabolism**	PATH:ko00620	2.1	2.1	1.8	2.2	2.0	1.7	2.3	4.8	3.5	1.9	2.2	2.5	3.1	3.0	2.2	1	1	3	2	1	1	1	2	1	1	1	1	1	1	1
**Secretion system**	BR:ko02044	1.1	1.0	0.9	0.7	1.4	0.7	0.8	1.9	1.6	1.9	0.8	0.7	1.9	1.8	1.7	1	1	2	2	1	1	1	2	3	1	1	1	1	1	1
**Transporters**	BR:ko02000	3.8	4.8	5.3	6.1	3.0	5.3	7.2	13.0	10.7	6.2	4.3	6.9	11.1	9.1	7.7	1	1	2	2	2	1	1	2	1	3	1	1	1	1	1
**Fructose and mannose metabolism**	PATH:ko00051	0.6	0.5	0.5	0.5	0.7	0.6	0.6	1.1	0.9	0.5	0.7	0.6	0.8	0.8	0.5	1	1	2	2	1	1	1	2	1	1	1	1	1	1	1
**Pentose phosphate pathway**	PATH:ko00030	1.0	0.8	0.8	0.9	0.9	0.8	0.8	1.5	1.6	1.0	1.0	0.8	1.3	1.4	1.2	1	1	2	2	1	1	1	2	2	1	1	1	1	1	1
**Nitrogen metabolism**	PATH:ko00910	0.8	1.2	1.3	1.4	1.0	1.0	1.5	0.9	1.2	1.3	0.9	1.2	0.8	1.4	2.0	1	1	1	1	1	1	1	1	1	2	1	1	1	1	2
**Glyoxylate and dicarboxylate metabolism**	PATH:ko00630	5.5	1.6	4.4	1.3	1.5	10.4	2.0	4.3	1.1	0.7	6.6	2.1	2.8	0.7	0.9	2	1	1	1	0	1	1	1	1	1	1	1	1	1	1
**Sulfur metabolism**	PATH:ko00920	0.7	0.9	1.6	1.0	0.5	0.6	1.5	1.6	1.1	0.7	0.8	1.7	2.1	0.7	0.6	1	2	1	1	1	1	2	1	1	1	1	1	1	1	1
**Type I diabetes mellitus**	PATH:ko04940	3.5	1.3	2.9	1.2	0.6	3.6	2.2	2.4	0.7	0.3	5.4	2.4	2.2	0.4	0.4	1	2	1	1	0	2	2	1	0	1	2	1	1	1	1
**Carbon fixation in photosynthetic organisms**	PATH:ko00710	6.0	1.8	4.6	1.5	1.5	11.1	1.7	5.1	1.8	1.0	7.3	2.2	3.3	1.2	1.0	2	1	1	1	1	1	1	1	1	1	1	1	1	1	1
**Plant-pathogen interaction**	PATH:ko04626	3.3	2.3	3.1	2.9	1.4	5.9	4.2	2.7	1.9	1.3	5.0	4.1	2.2	1.4	0.7	2	2	1	1	1	2	2	1	0	1	1	1	1	1	1
**Translation factors**	BR:ko03012	4.7	3.1	3.8	3.5	2.3	6.8	5.3	3.2	2.5	1.9	6.5	5.5	2.6	2.5	1.9	1	2	1	1	1	1	2	1	1	1	1	1	1	1	1
**Methane metabolism**	PATH:ko00680	0.6	0.8	0.9	1.0	0.8	0.8	1.4	0.6	0.5	0.4	0.7	1.2	0.4	0.4	0.6	1	2	1	0	0	1	1	0	0	1	1	1	1	1	1
**beta-Alanine metabolism**	PATH:ko00410	0.5	0.7	0.8	1.1	0.9	0.8	1.2	0.5	0.4	0.3	0.7	1.0	0.4	0.3	0.6	2	2	1	0	0	1	1	1	0	1	1	1	1	1	2
**Selenoamino acid metabolism**	PATH:ko00450	1.1	1.3	2.2	1.3	1.2	0.8	2.0	1.8	1.1	0.5	1.1	2.1	2.2	0.7	0.6	1	2	1	1	0	1	2	1	1	1	1	1	1	1	1
**Aminoacyl-tRNA biosynthesis**	PATH:ko00970	1.0	1.1	0.9	1.0	1.7	0.4	0.9	1.2	1.5	1.7	0.7	0.9	1.0	1.6	1.9	0	1	1	2	1	1	1	1	2	1	2	1	1	1	1
**Cysteine and methionine metabolism**	PATH:ko00270	1.5	1.6	2.4	1.7	1.3	1.2	2.3	2.0	1.3	0.8	1.4	2.3	2.2	0.8	1.0	1	1	1	1	1	1	1	1	0	1	1	1	1	1	1
**RNA degradation**	PATH:ko03018	6.3	2.9	4.3	2.2	2.1	5.4	3.7	3.4	1.7	1.6	7.9	4.1	3.5	1.2	1.5	1	1	1	1	1	1	1	1	1	1	1	1	1	1	1
**Fatty acid biosynthesis**	PATH:ko00061	0.9	0.8	0.6	0.8	0.7	0.9	1.0	0.9	0.8	0.5	1.0	1.1	0.6	0.8	0.8	1	1	1	1	1	1	1	1	1	1	1	1	1	1	2
**Photosynthesis proteins**	BR:ko00194	10.5	6.5	10.0	7.5	7.4	14.4	6.3	6.0	4.2	1.9	11.7	6.5	5.7	2.0	1.5	1	1	1	1	0	1	1	1	0	0	1	1	1	0	1
**Chromosome**	BR:ko03036	1.0	1.2	1.1	1.4	1.6	0.6	1.5	1.2	1.7	1.5	1.0	1.3	1.3	1.8	1.8	1	1	1	1	1	1	1	1	1	1	2	1	1	1	1
**Chaperones and folding catalysts**	BR:ko03110	7.8	4.1	8.8	3.2	5.2	7.3	5.2	5.7	2.6	2.4	10.0	5.1	6.3	2.1	2.7	1	1	1	1	0	1	1	1	1	1	1	1	1	1	1
**Photosynthesis**	PATH:ko00195	9.5	5.9	9.5	5.8	7.0	11.9	5.4	5.6	3.8	1.9	10.1	5.4	5.5	2.0	1.4	1	1	1	1	0	1	1	1	0	0	1	1	1	1	1
**Arginine and proline metabolism**	PATH:ko00330	1.1	1.4	1.6	1.6	1.4	1.2	1.7	0.8	0.9	1.0	1.2	1.6	0.8	1.0	1.3	1	1	1	1	1	1	1	0	1	1	1	1	1	1	1
**Alanine, aspartate and glutamate metabolism**	PATH:ko00250	1.1	1.4	1.5	1.3	1.6	1.1	1.5	0.9	1.0	1.3	1.1	1.4	0.9	1.3	1.9	1	1	1	1	1	1	1	1	1	1	1	1	1	1	1
**Reductive carboxylate cycle (CO2 fixation)**	PATH:ko00720	0.9	1.4	1.0	1.1	1.2	0.7	1.5	0.8	0.9	1.1	0.8	1.2	0.8	0.9	1.4	1	1	1	1	1	1	1	1	1	1	1	1	1	1	1
**Cell cycle - Caulobacter**	PATH:ko04112	0.8	1.1	1.3	1.0	1.4	0.7	1.2	1.3	1.0	0.8	0.8	1.0	1.4	1.1	1.2	1	1	1	1	1	1	1	1	1	1	1	1	1	1	2
**Ribosome**	PATH:ko03010	7.8	5.9	5.0	4.4	5.6	5.9	5.8	2.8	2.8	3.8	7.3	6.5	3.1	3.1	5.0	1	1	1	1	1	1	1	1	1	1	1	1	1	1	1
**Ribosome**	BR:ko03011	7.8	5.9	5.0	4.4	5.6	5.9	5.8	2.8	2.8	3.8	7.3	6.5	3.1	3.1	5.0	1	1	1	1	1	1	1	1	1	1	1	1	1	1	1
**RNA polymerase**	PATH:ko03020	2.8	2.9	2.1	2.6	2.1	2.2	3.2	1.2	1.4	1.2	2.8	2.9	1.2	1.4	2.1	1	1	1	1	1	1	1	1	1	1	1	1	1	1	2
**Purine metabolism**	PATH:ko00230	5.2	5.0	3.6	4.4	4.0	3.7	4.9	2.6	3.2	3.3	4.9	5.1	2.8	3.2	4.2	1	1	1	1	1	1	1	1	1	1	1	1	1	1	1
**Citrate cycle (TCA cycle)**	PATH:ko00020	1.6	2.1	1.5	1.8	2.0	1.3	2.1	1.4	1.5	1.4	1.6	1.9	1.4	1.4	2.0	1	1	1	1	1	1	1	1	1	1	1	1	1	1	1
**Oxidative phosphorylation**	PATH:ko00190	3.0	5.4	4.6	4.4	4.8	3.1	5.7	2.8	2.5	2.4	2.7	4.1	2.6	2.4	3.3	1	1	1	1	1	1	1	1	1	1	1	1	1	1	1
**DNA repair and recombination proteins**	BR:ko03400	3.9	4.5	3.5	4.1	4.3	2.7	4.7	2.1	3.0	3.4	3.5	4.4	2.3	3.2	4.0	1	1	1	1	1	1	1	1	1	1	1	1	1	1	1
**Pyrimidine metabolism**	PATH:ko00240	4.6	4.3	3.4	3.7	3.5	3.3	4.4	2.2	2.6	2.7	4.2	4.5	2.2	2.3	3.1	1	1	1	1	1	1	1	1	1	1	1	1	1	1	1
**Peptidases**	BR:ko01002	3.1	2.6	4.8	2.4	3.9	2.7	2.6	2.6	1.8	2.3	3.1	2.6	3.3	1.9	2.0	1	1	1	1	1	1	1	1	1	1	1	1	1	1	1
**DNA replication proteins**	BR:ko03032	0.9	1.2	0.8	1.0	1.6	0.5	1.1	0.7	0.9	0.9	0.8	1.1	0.8	1.0	1.4	1	1	1	1	1	1	1	1	1	1	1	1	1	1	1
**Glycine, serine and threonine metabolism**	PATH:ko00260	1.1	1.6	1.5	1.6	1.6	0.7	1.5	0.8	0.9	1.0	0.9	1.2	0.8	0.9	1.4	1	1	1	1	1	1	1	1	1	1	1	1	1	1	2
**Porphyrin and chlorophyll metabolism**	PATH:ko00860	1.5	1.9	0.8	1.6	1.5	0.6	1.2	0.5	1.2	1.0	1.4	1.4	0.7	1.0	1.2	0	1	1	1	1	1	1	1	1	1	2	1	2	1	1
**Lipid biosynthesis proteins**	BR:ko01004	1.2	1.5	1.0	1.3	1.3	1.0	1.4	0.9	1.1	1.1	1.0	1.2	0.8	1.1	1.2	1	1	1	1	1	1	1	1	1	1	1	1	1	1	1
**Valine, leucine and isoleucine biosynthesis**	PATH:ko00290	1.3	1.4	1.0	1.0	1.4	0.9	1.2	0.6	0.8	0.9	1.2	1.3	0.8	0.8	1.2	1	1	1	1	1	1	1	1	1	1	1	1	1	1	1

Table S6. Selected K0 orthologs whose expression changes significantly in the amendment transcriptomic datasets relative to the no-amendment control

Significant ko from AC test with >5x increase treatment/ctl (either B3, B2, or both)

Change values that are not significant (FDR >0.05) are in grey

FOLD CHANGE (% treatment / % control)

		+Glc+N/control (B2/B1)					+Glc+N+MPn/control (B3/B1)					+Glc+N+MPn/+Glc+N (B3/B2)				
**Kegg_ko**	**Description**	**0 h**	**12 h**	**24 h**	**36 h**	**48 h**	**0 h**	**12 h**	**24 h**	**36 h**	**48 h**	**0 h**	**12 h**	**24 h**	**36 h**	**48 h**
**PTS systems**																
K08483	pstI; phosphotransferase system, enzyme I, PtsI	1.0	0.5	87.0	18.8	28.3	0.6	1.0	53.7	33.3	18.7	0.7	1.8	0.6	1.8	0.7
K02784	hpr; phosphotransferase system phosphocarrier protein HPr	1.0	0.5	15.3	17.1	36.8	0.6	0.5	20.7	8.3	9.4	0.7	0.9	1.4	0.5	0.3
K02777	crr; PTS system, glucose-specific IIA component PTS-Glc-EIIA	1.0	0.5	2063.2	186.8	8.5	0.6	0.5	1591.8	39.2	2.6	0.7	0.9	0.8	0.2	0.3
K02778	ptsG; PTS system, glucose-specific IIB component	1.0	0.5	63.5	4.3	36.8	0.6	0.5	36.5	13.1	21.8	0.7	0.9	0.6	3.1	0.6
K02803	nagE; PTS system, N-acetylglucosamine-specific IIB component	1.0	0.5	30.6	4.3	11.3	0.6	0.5	19.3	3.6	9.4	0.7	0.9	0.6	0.8	0.8
K02809	scrA; PTS system, sucrose-specific IIBC component	1.0	0.5	0.6	0.2	1.4	0.6	0.5	0.3	0.3	15.6	0.7	0.9	0.6	1.8	11.0
**Polyhydroxyalkanoate metabolism**																
K00023	phbB; acetoacetyl-CoA reductase	3.8	5.5	697.5	122.4	93.5	0.6	1.0	551.5	94.9	35.9	0.2	0.2	0.8	0.8	0.4
K00626	phbA, atoB; acetyl-CoA C-acetyltransferase	1.3	0.7	19.7	9.3	1.1	0.8	1.7	10.0	8.1	1.0	0.6	2.5	0.5	0.9	0.9
K03821	phbC, phaC; polyhydroxyalkanoate synthase	1.0	0.3	130.6	16.2	42.5	0.3	0.5	115.7	5.6	40.5	0.3	1.8	0.9	0.3	1.0
**Amino sugar metabolism**																
K01183	class III chitinase	1.0	0.5	0.2	0.7	22.7	0.6	0.5	0.2	2.4	15.6	0.7	0.9	1.2	3.6	0.7
K02803	nagE; PTS system, N-acetylglucosamine-specific IIB component	1.0	0.5	30.6	4.3	11.3	0.6	0.5	19.3	3.6	9.4	0.7	0.9	0.6	0.8	0.8
K00884	nagK; N-acetyl-D-glucosamine kinase	1.0	0.5	4.7	2.6	14.2	0.6	0.5	12.4	8.3	12.5	0.7	0.9	2.6	3.2	0.9
K02564	nagB; glucosamine-6-phosphate deaminase	0.5	0.3	1.8	0.2	22.7	0.3	0.3	0.3	3.0	1.6	0.7	0.9	0.2	18.1	0.1
K00886	ppgK; polyphosphate glucokinase	1.0	0.3	0.6	0.2	11.3	0.6	0.3	0.3	0.3	1.6	0.7	0.9	0.6	1.8	0.1
K00845	glk; glucokinase	0.5	0.1	0.1	0.3	0.7	1.2	0.1	0.1	0.2	15.6	2.6	1.8	1.2	0.7	22.0
K01835	pgm; phosphoglucomutase	0.7	0.5	8.6	2.4	2.6	0.3	3.0	10.0	2.1	1.2	0.4	5.5	1.2	0.9	0.5
K00963	UGP2, galU, galF; UTP--glucose-1-phosphate uridylyltransferase	1.0	0.5	30.6	6.2	0.8	3.7	1.0	26.2	4.2	2.3	3.9	1.8	0.9	0.7	2.9
K02777	crr; PTS system, glucose-specific IIA component PTS-Glc-EIIA	1.0	0.5	2063.2	186.8	8.5	0.6	0.5	1591.8	39.2	2.6	0.7	0.9	0.8	0.2	0.3
K02778	ptsG; PTS system, glucose-specific IIB component	1.0	0.5	63.5	4.3	36.8	0.6	0.5	36.5	13.1	21.8	0.7	0.9	0.6	3.1	0.6
**Starch**																
K01176	amyA, malS; alpha-amylase [EC:3.2.1.1]	1.0	0.5	1.2	5.6	31.2	0.6	0.5	5.5	8.3	15.6	0.7	0.9	4.7	1.5	0.5
K01187	alpha-glucosidase (EC:3.2.1.-); K01187 alpha-glucosidase [EC:3.2.1.20]	0.2	0.1	2.4	0.8	0.7	0.3	0.1	2.1	0.3	12.5	1.3	0.9	0.9	0.4	17.6
K02777	crr; PTS system, glucose-specific IIA component PTS-Glc-EIIA	1.0	0.5	2063.2	186.8	8.5	0.6	0.5	1591.8	39.2	2.6	0.7	0.9	0.8	0.2	0.3
K02778	ptsG; PTS system, glucose-specific IIB component	1.0	0.5	63.5	4.3	36.8	0.6	0.5	36.5	13.1	21.8	0.7	0.9	0.6	3.1	0.6
**Phosphate metabolism**																
K02040	pstS; phosphate transport system substrate-binding protein	0.2	0.8	70.5	90.4	45.5	0.7	1.2	71.1	53.3	10.7	3.5	1.6	1.0	0.6	0.2
K02037	pstC; phosphate transport system permease protein	0.6	0.8	43.8	101.5	34.4	0.2	0.1	31.3	189.1	22.2	0.3	0.2	0.7	1.9	0.6
K02038	pstA; phosphate transport system permease protein	0.2	0.5	3.2	4.4	2.7	0.9	0.2	4.7	9.1	2.9	4.6	0.3	1.5	2.1	1.1
K02036	pstB; phosphate transport system ATP-binding protein	0.2	0.2	2.2	18.8	26.9	0.9	0.1	4.7	25.6	16.4	3.9	0.5	2.1	1.4	0.6
K01077	phoA, phoB; alkaline phosphatase	0.5	0.5	10.9	50.4	12.4	1.9	0.5	6.8	30.2	8.6	3.9	0.9	0.6	0.6	0.7
K02039	phoU; phosphate transport system protein	3.8	0.5	0.3	0.6	5.7	1.2	0.5	0.8	0.2	31.2	0.3	0.9	2.6	0.3	5.5
K07657	phoB; two-component system phosphate regulon response regulator	0.5	0.1	1.2	17.1	1.8	0.3	0.1	2.2	16.7	2.1	0.7	0.9	1.8	1.0	1.2
K07636	phoR; two-component system phosphate regulon sensor histidine kin	1.0	0.5	6.5	7.7	5.2	0.6	0.5	3.3	8.6	7.3	0.7	0.9	0.5	1.1	1.4
K07093	phoX; alkaline phosphatase PhoX	1.0	0.5	10.6	49.7	87.1	1.2	0.5	14.8	112.4	17.2	1.3	0.9	1.4	2.3	0.2
**Nitrogen metabolism**																
K00362	nirB; nitrite reductase (NAD(P)H) large subunit	1.0	0.5	5.9	0.7	48.2	0.6	0.5	8.3	8.3	196.5	0.7	0.9	1.4	12.6	4.1
K00363	nirD; nitrite reductase (NAD(P)H) small subunit	1.0	0.5	3.5	1.0	39.7	0.6	0.5	1.4	0.6	15.6	0.7	0.9	0.4	0.6	0.4
K00372	nasA; nitrate reductase catalytic subunit [EC:1.7.99.4]	1.0	0.3	7.1	4.0	38.3	0.6	0.3	10.3	8.3	190.2	0.7	0.9	1.5	2.1	5.0

K00260	gudB, rocG; glutamate dehydrogenase	1.9	0.5	8.8	9.6	3.5	0.6	1.0	9.3	11.3	2.9	0.3	1.8	1.1	1.2	0.8
**Two-component systems**																
K07657	phoB; two-component system phosphate regulon response regulator	0.5	0.1	1.2	17.1	1.8	0.3	0.1	2.2	16.7	2.1	0.7	0.9	1.8	1.0	1.2
K07636	phoR; two-component system phosphate regulon sensor histidine kina	1.0	0.5	6.5	7.7	5.2	0.6	0.5	3.3	8.6	7.3	0.7	0.9	0.5	1.1	1.4
K07639	rstB; stress response two-component system sensor histidine kinase R	0.5	0.5	0.6	1.3	31.2	0.3	0.5	1.4	1.8	3.1	0.7	0.9	2.3	1.4	0.1
K07661	rstA; stress response two-component system response regulator Rst	1.0	0.3	1.2	3.0	19.8	0.6	0.3	2.1	1.8	1.6	0.7	0.9	1.8	0.6	0.1
K07678	barA; central carbon metabolism two-component system sensor histic	1.9	0.5	1.2	1.3	8.5	0.6	0.5	1.4	6.5	4.7	0.3	0.9	1.2	5.0	0.6
K07689	uvrY; central carbon metabolism two-component system transcriptior	1.0	0.3	356.4	250.7	62.3	0.6	0.3	451.0	80.9	1.6	0.7	0.9	1.3	0.3	0.0
K10910	LuxP; autoinducer 2-binding periplasmic protein LuxP	1.0	0.5	4.7	12.8	17.0	0.6	0.5	16.5	16.1	1.6	0.7	0.9	3.5	1.2	0.1
K10909	luxQ; two-component system, autoinducer 2 sensor kinase/phosphata	1.0	0.5	7.1	3.3	22.7	0.6	0.5	4.8	7.7	3.1	0.7	0.9	0.7	2.3	0.1
K10912	luxO; two-component system, repressor protein LuxO	1.0	0.5	54.1	24.1	11.3	0.6	0.5	20.0	9.5	4.7	0.7	0.9	0.4	0.4	0.4
K10125	dctB; two-component system, C4-dicarboxylate transport sensor histic	1.0	0.5	1.2	2.0	48.2	2.5	0.5	6.9	5.4	28.1	2.6	0.9	5.9	2.7	0.6
K10126	dctD; two-component system, C4-dicarboxylate transport response re	1.0	1.1	2.4	3.3	14.2	0.6	1.0	1.4	1.8	21.8	0.7	0.9	0.6	0.5	1.5
K13598	NtrY; nitrogen regulation two-component signal transduction histidine	1.0	1.1	0.2	0.2	0.1	0.6	0.5	0.1	0.3	2.5	0.7	0.5	0.6	1.8	17.6
K13599	NtrX; two component, nitrogen regulation response regulator NtrX	1.9	3.8	0.1	0.1	0.2	0.6	0.5	0.1	0.3	3.1	0.3	0.1	1.2	3.6	17.6
K10942	flrB; signal transduction histidine kinase; flagellum biosynthesis FlrB	1.0	0.5	14.1	8.9	7.1	0.6	0.5	7.9	11.3	3.1	0.7	0.9	0.6	1.3	0.4
K07773	ArcA; aerobic respiration two-component response regulator ArcA	1.0	0.5	4.7	5.3	7.8	0.6	0.5	11.0	7.7	0.8	0.7	0.9	2.3	1.5	0.1
**Chemotaxis**																
K03407	cheA; chemotaxis two-component system sensor kinase CheA	0.5	0.5	4.7	4.3	22.0	0.3	0.3	2.9	11.6	14.0	0.7	0.5	0.6	2.7	0.6
K03415	cheV; chemotaxis two-component system response regulator CheV	1.0	0.3	3.5	16.1	35.4	0.6	0.3	7.6	14.3	7.8	0.7	0.9	2.1	0.9	0.2
K03413	cheY; chemotaxis regulator CheY, transmits signal to flagellar motor	1.0	0.5	6.5	6.3	11.3	1.2	0.5	3.4	9.5	12.5	1.3	0.9	0.5	1.5	1.1
K03406	mcp; methyl-accepting chemotaxis protein	3.8	1.1	12.0	27.6	18.2	1.2	0.3	14.3	36.9	6.1	0.3	0.2	1.2	1.3	0.3
K00575	cheR; methylase of chemotaxis methyl-accepting protein CheR	0.5	0.3	6.3	24.1	18.4	0.3	0.3	5.9	28.0	6.2	0.7	0.9	0.9	1.2	0.3
K03412	cheB; two-component system, response regulator CheB	1.0	0.5	38.8	2.0	2.5	0.6	0.5	21.3	7.7	7.4	0.7	0.9	0.5	3.9	3.0
K03408	cheW; purine-binding chemotaxis protein CheW	1.9	0.3	20.0	9.9	25.5	1.2	0.3	22.7	6.5	25.0	0.7	0.9	1.1	0.7	1.0
**Flagellar assembly and regulation**																
K02388	flgC; flagellar basal body rod protein FlgC	1.0	0.3	2.9	4.3	2.0	1.2	0.1	3.4	26.8	0.3	1.3	0.5	1.2	6.2	0.2
K02391	flgF; flagellar basal-body rod protein FlgF	0.5	0.5	2.4	4.6	4.3	0.3	0.5	9.6	13.7	2.1	0.7	0.9	4.1	3.0	0.5
K02394	flgI; flagellar basal body P-ring protein FlgI	0.5	2.2	1.8	4.0	6.2	0.3	0.3	2.1	10.7	3.4	0.7	0.1	1.2	2.7	0.6
K02395	flgJ; flagellar rod assembly protein/muramidase FlgJ	1.9	0.3	2.4	5.3	17.0	0.6	0.3	9.6	11.3	3.1	0.3	0.9	4.1	2.1	0.2
K02397	flgL; flagellar hook-associated protein FlgL	1.0	0.5	3.5	1.2	7.1	0.6	0.1	11.0	3.0	4.7	0.7	0.2	3.1	2.6	0.7
K02396	flgK; flagellar hook-associated protein FlgK	2.9	0.2	4.1	1.6	14.2	0.3	0.1	9.3	3.1	0.8	0.1	0.9	2.3	2.0	0.1
K02399	flgN; flagella synthesis protein FlgN	0.2	0.3	5.9	5.3	28.3	0.2	0.3	5.5	7.7	1.6	0.7	0.9	0.9	1.5	0.1
K02414	fliK; polar flagellar hook-length control protein FliK	1.0	0.5	16.5	8.6	42.5	0.6	0.5	13.1	23.8	18.7	0.7	0.9	0.8	2.8	0.4
K02422	fliS; flagellar protein FliS	0.5	0.5	1.5	4.6	3.2	0.3	0.5	2.1	17.2	0.2	0.7	0.9	1.4	3.7	0.1
K10941	flrA; sigma-54 specific flagellar transcriptional regulator	0.6	0.5	6.5	1.6	8.5	0.1	2.0	2.8	3.7	4.7	0.2	3.6	0.4	2.4	0.6
K10942	flrB, two-component system, sensor histidine kinase FlrB	1.0	0.5	14.1	8.9	7.1	0.6	0.5	7.9	11.3	3.1	0.7	0.9	0.6	1.3	0.4
K02405	fliA; flagellar biosynthesis sigma factor FliA	0.5	0.5	20.0	12.2	4.3	0.6	4.0	16.5	19.0	1.3	1.3	7.3	0.8	1.6	0.3
K02404	flhF; flagellar biosynthesis regulator FlhF	0.5	0.5	5.3	11.5	19.4	0.3	1.0	3.4	22.6	5.2	0.7	1.8	0.7	2.0	0.3
**ABC Transporters**																
K02040	pstS; phosphate ABC transporter, periplasmic binding protein	0.2	0.8	70.5	90.4	45.5	0.7	1.2	71.1	53.3	10.7	3.5	1.6	1.0	0.6	0.2
K02037	pstC; phosphate ABC transporter, permease protein	0.6	0.8	43.8	101.5	34.4	0.2	0.1	31.3	189.1	22.2	0.3	0.2	0.7	1.9	0.6
K02038	pstA; phosphate ABC transporter, permease protein	0.2	0.5	3.2	4.4	2.7	0.9	0.2	4.7	9.1	2.9	4.6	0.3	1.5	2.1	1.1
**K02044**	**phnD; phosphonate ABC transporter, periplasmic binding protein**	0.1	0.7	0.6	0.6	1.1	0.4	0.2	0.3	0.1	13.3	3.9	0.3	0.6	0.1	12.5

**K02042**	**phnE; phosphonate ABC transporter permease**	0.5	0.8	0.6	0.2	0.9	0.3	0.3	0.3	2.4	29.1	0.7	0.3	0.6	14.4	30.8
**K02041**	**phnC; phosphonate ABC transporter, ATP-binding protein**	1.0	0.3	2.4	0.3	8.5	0.3	1.0	2.8	0.1	102.9	0.3	3.6	1.2	0.5	12.1
K05813	ugpB; glycerol 3-phosphate transport system substrate-binding protei	0.3	0.1	0.6	3.6	12.8	0.3	0.5	0.2	0.9	5.2	1.0	3.6	0.4	0.2	0.4
K05814	ugpA; glycerol-3-phosphate ABC transporter permease protein	1.0	0.5	2.4	12.5	25.5	0.6	0.5	0.3	0.6	6.2	0.7	0.9	0.1	0.0	0.2
K05815	ugpE; glycerol 3-phosphate transport system permease protein	1.0	0.5	1.2	5.3	34.0	0.6	0.5	2.8	2.4	12.5	0.7	0.9	2.3	0.5	0.4
K10108	malE; maltose ABC transporter periplasmic protein	1.0	1.1	9.4	10.9	28.3	0.6	0.5	11.5	13.1	1.6	0.7	0.5	1.2	1.2	0.1
K10109	malF; maltose transporter permease protein	1.0	0.5	14.1	4.6	5.7	0.6	1.0	25.5	3.6	3.1	0.7	1.8	1.8	0.8	0.6
K10110	malG; maltose transporter permease	1.0	0.5	16.5	3.3	8.5	0.6	0.5	17.9	4.2	6.2	0.7	0.9	1.1	1.3	0.7
K10111	maltose/maltodextrin transport system ATP-binding protein	1.9	0.3	5.9	4.6	6.4	1.2	1.0	12.4	7.1	2.3	0.7	3.6	2.1	1.5	0.4
K10543	xylF; D-xylose ABC transporter substrate-binding protein	1.0	0.5	1.2	0.2	1.4	0.6	1.0	0.7	0.3	84.2	0.7	1.8	0.6	1.8	59.4
K10544	xylH; D-xylose transport system permease protein	1.0	0.5	0.6	0.3	1.4	1.2	0.5	0.3	0.3	31.2	1.3	0.9	0.6	0.9	22.0
**Twin arginine translocation pathway (TAT)**																
K03117	tatB; sec-independent protein translocase protein TatB	1.0	1.1	481.1	126.5	9.9	0.3	2.0	383.5	76.1	7.8	0.3	1.8	0.8	0.6	0.8
K03116	tatA; sec-independent protein translocase protein TatA	1.0	0.3	10.9	3.9	1.1	0.6	0.3	8.1	2.7	0.4	0.7	0.9	0.7	0.7	0.4
**Others**																
K06194	lipoprotein NlpD	1.0	0.5	85.6	467.8	15.6	0.6	0.5	103.6	218.8	3.9	0.7	0.9	1.2	0.5	0.3
K12515	Flp pilus assembly protein TadG	1.0	0.5	3.5	7.6	36.8	0.6	0.5	9.0	5.9	3.1	0.7	0.9	2.5	0.8	0.1
**Alkylphosphonate metabolism**																
**K02041**	**phnC; phosphonate ABC transporter, ATP-binding protein**	1.0	0.3	2.4	0.3	8.5	0.3	1.0	2.8	0.1	102.9	0.3	3.6	1.2	0.5	12.1
**K02044**	**phnD; phosphonate ABC transporter, periplasmic binding protein**	0.1	0.7	0.6	0.6	1.1	0.4	0.2	0.3	0.1	13.3	3.9	0.3	0.6	0.1	12.5
**K02042**	**phnE; phosphonate ABC transporter permease**	0.5	0.8	0.6	0.2	0.9	0.3	0.3	0.3	2.4	29.1	0.7	0.3	0.6	14.4	30.8
**K02043**	**phnF; phosphonate transport system regulatory protein**	1.0	0.5	0.6	0.2	1.4	0.6	0.5	0.3	0.3	12.5	0.7	0.9	0.6	1.8	8.8
**K06166**	**phnG; alkylphosphonate utilization protein PhnG**	1.0	0.5	0.6	0.2	1.4	0.6	0.5	0.7	0.6	21.8	0.7	0.9	1.2	3.6	15.4
**K06165**	**phnH; carbon-phosphorus lyase complex subunit PhnH**	1.0	0.5	0.6	0.2	1.4	0.6	0.5	0.3	0.6	21.8	0.7	0.9	0.6	3.6	15.4
**K06164**	**phnI; phosphonate metabolism protein PhnI**	1.0	0.5	0.6	0.2	1.4	0.6	0.5	0.3	0.3	146.6	0.7	0.9	0.6	1.8	103.5
**K06163**	**phnJ; phosphonate metabolism protein PhnJ**	1.0	0.5	0.1	0.2	8.5	0.6	0.5	0.1	0.6	99.8	0.7	0.9	0.6	3.6	11.7
**K05781**	**phnK; phosphonate C-P lyase system protein PhnK**	1.0	0.5	0.6	0.2	1.4	0.6	0.5	0.3	0.3	249.5	0.7	0.9	0.6	1.8	176.1
**K05780**	**phnL; carbon-phosphorus lyase complex subunit PhnL**	3.8	0.5	0.6	0.3	1.4	0.6	0.5	0.7	0.3	174.7	0.2	0.9	1.2	0.9	123.3
**K06162**	**phnM; phosphonate metabolism protein PhnM**	1.0	0.5	1.2	0.2	5.7	0.6	0.5	0.3	0.3	286.9	0.7	0.9	0.3	1.8	50.6
**K05774**	**phnN; 1,5-bisphosphokinase (PRPP-forming) PhnN**	1.0	0.5	0.6	0.2	1.4	0.6	0.5	0.3	0.3	46.8	0.7	0.9	0.6	1.8	33.0
**K06167**	**phnP; carbon-phosphorus lyase complex accessory protein**	1.0	0.3	1.2	0.2	0.4	0.6	0.3	2.8	0.6	6.2	0.7	0.9	2.3	3.6	17.6

Table S7. Change in sRNA expression in treatment metatranscriptoms relative to the no-amendement control

		+Glc+N/control (B2/B1)*					+Glc+N+MPn/control (B3/B1)					
**Rfam family**	**Annotation**	**0 h**	**12 h**	**24 h**	**36 h**	**48 h**	**0 h**	**12 h**	**24 h**	**36 h**	**48 h**	**max chang**
RF00018	CsrB/RsmB RNA family	1	0	2244	4076	204	1	0	1537	4375	11	4375
RF01766	cspA thermoregulator	3	1	70	33	2	1	0	86	26	2	86
RF00630	Pseudomonas sRNA P26	1	1	45	23	13	1	0	70	48	16	70
RF00017	Eukaryotic type signal recognition particle RNA	0	1	6	0	0	0	8	1	0	68	68
RF01417	Retroviral 3'UTR stability element	1	1	0	0	2	2	4	1	0	51	51
RF00168	Lysine riboswitch	1	1	1	23	46	1	1	34	32	4	46
RF01854	Bacterial large signal recognition particle RNA	0	0	0	0	0	0	0	0	1	37	37
RF00504	Glycine riboswitch	1	1	0	20	1	1	1	2	35	0	35
RF00127	t44 RNA	1	1	2	12	27	1	1	8	32	1	32
RF00140	Alpha operon ribosome binding site	1	0	19	13	20	1	0	24	20	5	24
RF00391	RtT RNA	1	0	2	1	13	1	0	1	23	19	23
RF00378	Qrr RNA	1	1	4	0	23	1	1	6	2	1	23
RF01086	Long range pseudoknot	2	1	5	17	3	2	0	5	17	1	17
RF00162	SAM riboswitch (S box leader)	1	1	1	0	2	1	1	1	1	17	17
RF00100	7SK RNA	0	1	4	0	2	0	4	1	0	16	16
RF01055	Moco (molybdenum cofactor) riboswitch	1	1	6	5	8	1	1	11	16	0	16
RF00004	U2 spliceosomal RNA	0	1	16	2	0	0	7	3	0	1	16
RF00375	HIV primer binding site (PBS)	0	2	2	0	2	0	9	0	0	14	14
RF01808	MicX Vibrio cholerae sRNA	1	1	6	4	2	1	1	14	1	3	14
RF01770	Enterobacteria rnk leader	1	1	4	13	2	1	1	8	1	1	13

* Fold change = % of total non-rRNA reads in treatment / % of total non-rRNA reads in no amendemnt control
Families with no assigned reads where given 0.5 reads for the fold change calculations

Table S8. Annotation of fosmids found in MPn-degradation screen

B3TF_MPn_2 Annotation (34418 bp)

ORF	Gene	Strand	Start	End	Hit-Name	Description	E-Value	%-Ident	COG	Description
1		+	2	871	ZP_08862186	glycosyl transferase, group 2 family protein [Ruegeria sp. TW15]	5.00E-86	58%	COG1216	Predicted glycosyltransferases
2		+	872	5443	ZP_08862187	non-ribosomal peptide synthetase [Ruegeria sp. TW15]	0	60%	COG1020	Non-ribosomal peptide synthetase modules and related proteins
3		+	5453	6121	ZP_01740674	phosphopantetheinyl transferase PptA, putative [Rhodobacterales bacterium HTCC2150]	4.00E-45	42%	COG2977	Phosphopantetheinyl transferase component of siderophore synthetase
4		+	6123	7130	ZP_00960957	hypothetical protein ISM_16720 [Roseovarius nubinhibens ISM]	3.00E-115	58%	SPO0845	hypothetical protein
5		+	7295	8578	ZP_05075001	lipopolysaccharide biosynthesis [Rhodobacterales bacterium HTCC2083]	2.00E-98	42%	SPO0840	chain length determinant protein, putative
6		-	8603	9697	ZP_05074144	exopolysaccharide biosynthesis domain protein [Rhodobacterales bacterium HTCC2083]	2.00E-90	51%	COG0489	ATPases involved in chromosome partitioning
7		-	9879	11957	ZP_01441594	oxidoreductase, Gfo/Idh/MocA family protein [Pelagibaca bermudensis HTCC2601]	0	64%	COG0673	Predicted dehydrogenases and related proteins
8		-	11969	12952	ZP_01878059	glycosyl transferase, group 2 family protein [Roseovarius sp. TM1035]	2.00E-144	67%	COG0463	Glycosyltransferases involved in cell wall biogenesis
9		-	12949	14214	ZP_05782847	glycosyl transferase, group 1 family [Citreicella sp. SE45]	0	66%	COG0438	Glycosyltransferase
10		-	14207	15256	ZP_01035530	oxidoreductase, Gfo/Idh/MocA family protein [Roseovarius sp. 217]	4.00E-59	42%	COG0451	Nucleoside-diphosphate-sugar epimerases
11		-	15285	16526	ZP_01441590	Oxidoreductase, N-terminal [Pelagibaca bermudensis HTCC2601]	0	65%	COG0673	Predicted dehydrogenases and related proteins
12		+	16622	17374	ZP_01740662	glycosyl transferase, WecB/TagA/CpsF family protein [Rhodobacterales bacterium HTCC2150]	5.00E-91	53%	COG1922	Teichoic acid biosynthesis proteins
13	*phnC*	+	17580	18383	ZP_01877756	Phosphonate ABC transporter PhnC, ATP-binding protein [Roseovarius sp. TM1035]	7.00E-162	83%	COG3638	ABC-type phosphate/phosphonate transport system, ATPase component
14	*phnD*	+	18450	19358	ZP_01755464	phosphonate ABC transporter, periplasmic phosphonate-binding protein [Roseobacter sp. SK209-2-6]	0	92%	COG3221	ABC-type phosphate/phosphonate transport system, periplasmic component
15	*phnE*	+	19424	20317	YP_611936	phosphonate ABC transporter permease [Ruegeria sp. TM1040]	5.00E-161	75%	COG3639	ABC-type phosphate/phosphonate transport system, permease component
16	*phnE*	+	20314	21612	ZP_05077436	phosphonate uptake transporter [Rhodobacterales bacterium Y4I]	0	77%	COG3639	ABC-type phosphate/phosphonate transport system, permease component
17		+	21697	22314	YP_611934	hexapaptide repeat-containing transferase [Ruegeria sp. TM1040]	2.00E-100	68%	COG0110	Acetyltransferase (isoleucine patch superfamily)
18	*phnM1*	-	22517	23680	ZP_01902017	alkylphosphonate utilization protein PhnM, putative [Roseobacter sp. AzwK-3b]	2.00E-176	65%	COG3454	Metal-dependent hydrolase involved in phosphonate metabolism
19	*phnF*	-	23806	24513	ZP_02151518	transcriptional regulator, GntR family protein [Oceanibulbus indolifex HEL-45]	7.00E-103	63%	COG2188	Transcriptional regulators
20	*phnG*	+	24619	25083	ZP_05049987	phosphonate C-P lyase system protein PhnG [Octadecabacter antarcticus 307]	2.00E-54	60%	COG3624	Uncharacterized enzyme of phosphonate metabolism
21	*phnH*	+	25083	25664	ZP_01902020	alkylphosphonate utilization protein PhnH [Roseobacter sp. AzwK-3b]	4.00E-77	64%	COG3625	Uncharacterized enzyme of phosphonate metabolism
22	*phnI*	+	25664	26773	ZP_02149153	carbon-phosphorus lyase complex subunit [Phaeobacter gallaeciensis 2.10]	0	86%	COG3626	Uncharacterized enzyme of phosphonate metabolism
24	*phnJ*	+	27302	28171	ZP_01036192	alkylphosphonate utilization protein PhnJ [Roseovarius sp. 217]	0	85%	COG3627	Uncharacterized enzyme of phosphonate metabolism
25	*phnK*	+	28168	28938	ZP_01004207	alkylphosphonate utilization protein PhnK [Loktanella vestfoldensis SKA53]	3.00E-166	91%	COG4107	ABC-type phosphonate transport system, ATPase component
26	*phnL*	+	28989	29687	ZP_00959408	alkylphosphonate utilization protein PhnL [Roseovarius nubinhibens ISM]	2.00E-127	83%	COG4778	ABC-type phosphonate transport system, ATPase component
27	*phnN*	+	29684	30241	ZP_02151525	Phosphonate metabolism, 1,5-bisphosphokinase (PRPP-forming) PhnN [Oceanibulbus indolifex HEL-45]	6.00E-55	56%	COG3709	Uncharacterized component of phosphonate metabolism
28	*rcsF*	-	30223	30906	ZP_02151526	hypothetical protein OIHEL45_01245 [Oceanibulbus indolifex HEL-45]	2.00E-90	59%	RSKD131	hypothetical protein
29	*phnM2*	+	30977	32119	YP_611924	phosphonate metabolism PhnM [Ruegeria sp. TM1040]	0	69%	COG3454	Metal-dependent hydrolase involved in phosphonate metabolism
30		+	32246	33127	ZP_05786253	aminoglycoside phosphotransferase [Silicibacter lacuscaerulensis ITI-1157]	6.00E-67	45%	SPO3464	hypothetical protein
31		-	33099	34418	YP_615021	2-octaprenylphenol hydroxylase [Ruegeria sp. TM1040]	0	77%	COG0661	Predicted unusual protein kinase

B3TF_MPn_8 Annotation (26216 bp)

ORF	Gene	Strand	Start	End	Hit-Name	Description	E-Value	%-Ident	COG	Description
1		+	3	2276	ZP_08731562	chitinase [Vibrio nigripulchritudo ATCC 27043]	0	52%	COG3979	Uncharacterized protein contain chitin-binding domain type 6
2	*phnC*	+	2540	3400	ZP_08731563	phosphonate/organophosphate ester transporter subunit [Vibrio nigripulchritudo ATCC 27043]	1.00E-142	86%	COG3638	ABC-type phosphate/phosphonate transport system, ATPase component
3	*phnD*	+	3416	4411	ZP_08731564	phosphonate/organophosphate ester transporter subunit PhnD [Vibrio nigripulchritudo ATCC 27043]	1.00E-169	90%	COG3221	ABC-type phosphate/phosphonate transport system, periplasmic component
4	*phnE*	+	4464	5276	ZP_08731565	hypothetical protein VINI7043_18079 [Vibrio nigripulchritudo ATCC 27043]	1.00E-137	91%	COG3639	ABC-type phosphate/phosphonate transport system, permease component
5	*phnF*	+	5321	6016	ZP_08731566	phosphonate metabolism transcriptional regulator PhnF [Vibrio nigripulchritudo ATCC 27043]	1.00E-125	93%	COG2188	Transcriptional regulators
6	*phnG*	+	6019	6474	ZP_08731567	PhnG protein [Vibrio nigripulchritudo ATCC 27043]	1.00E-69	83%	COG3624	Uncharacterized enzyme of phosphonate metabolism
7	*phnH*	+	6471	7088	ZP_08731568	phosphonate C-P lyase system protein PhnH [Vibrio nigripulchritudo ATCC 27043]	7.00E-74	68%	COG3625	Uncharacterized enzyme of phosphonate metabolism
8	*phnI*	+	7073	8191	ZP_08731569	phosphonate metabolism [Vibrio nigripulchritudo ATCC 27043]	0	84%	COG3626	Uncharacterized enzyme of phosphonate metabolism
9	*phnJ*	+	8175	9056	ZP_08731570	phosphonate metabolism PhnJ [Vibrio nigripulchritudo ATCC 27043]	1.00E-154	91%	COG3627	Uncharacterized enzyme of phosphonate metabolism
10	*phnK*	+	9050	9874	ZP_08731571	phosphonate C-P lyase system protein PhnK [Vibrio nigripulchritudo ATCC 27043]	1.00E-133	91%	COG4107	ABC-type phosphonate transport system, ATPase component
11	*phnL*	+	9879	10595	ZP_08731572	phosphonate C-P lyase system protein PhnL [Vibrio nigripulchritudo ATCC 27043]	1.00E-116	85%	COG4778	ABC-type phosphonate transport system, ATPase component
12	*phnM*	+	10609	11745	ZP_08731573	phosphonate metabolism protein PhnM [Vibrio nigripulchritudo ATCC 27043]	0	88%	COG3454	Metal-dependent hydrolase involved in phosphonate metabolism
13	*phnN*	+	11783	12340	ZP_08731574	ribose 1,5-bisphosphokinase [Vibrio nigripulchritudo ATCC 27043]	2.00E-64	68%	COG3709	Uncharacterized component of phosphonate metabolism
14	*phnP*	+	12331	13086	ZP_08731575	carbon-phosphorus lyase complex accessory protein [Vibrio nigripulchritudo ATCC 27043]	1.00E-115	78%	COG1235	Metal-dependent hydrolases of the beta-lactamase superfamily
15		+	13217	14197	ZP_08731576	alcohol dehydrogenase [Vibrio nigripulchritudo ATCC 27043]	1.00E-172	90%	COG0604	NADPH:quinone reductase and related Zn-dependent oxidoreductases
16		-	14316	15260	ZP_08731577	transcriptional regulator [Vibrio nigripulchritudo ATCC 27043]	1.00E-161	87%	COG0583	Transcriptional regulator
17		+	15765	16013	ZP_08731578	sulfur transfer protein SirA [Vibrio nigripulchritudo ATCC 27043]	2.00E-37	91%	COG0425	Predicted redox protein, regulator of disulfide bond formation
18		-	16387	16644	NP_932816	NADH dehydrogenase subunit II-related protein [Vibrio vulnificus YJ016]	2.00E-23	60%	VS_0036	NADH dehydrogenase subunit II-like protein
19		-	16733	17323	ZP_08731580	transmembrane protein [Vibrio nigripulchritudo ATCC 27043]	7.00E-87	88%	COG2119	Predicted membrane protein
20		+	17744	18664	ZP_01815889	glycyl-tRNA synthetase subunit alpha [Vibrionales bacterium SWAT-3]	1.00E-180	98%	COG0752	Glycyl-tRNA synthetase, alpha subunit
21		+	18667	20733	ZP_05943207	glycyl-tRNA synthetase beta chain [Vibrio orientalis CIP 102891 = ATCC 33934]	0	95%	COG0751	Glycyl-tRNA synthetase, beta subunit
22		-	20797	21165	NP_932812	hypothetical protein VV0019 [Vibrio vulnificus YJ016]	2.00E-20	38%	VV0019	hypothetical protein
23		-	21293	21655	NP_932812	hypothetical protein VV0019 [Vibrio vulnificus YJ016]	2.00E-28	52%	VV0019	hypothetical protein
24		+	21912	23288	ZP_08731585	valine--pyruvate transaminase [Vibrio nigripulchritudo ATCC 27043]	0	96%	COG3977	Alanine-alpha-ketoisovalerate (or valine-pyruvate) aminotransferase
25		-	23361	23795	ZP_08731587	heat shock protein [Vibrio nigripulchritudo ATCC 27043]	8.00E-75	94%	COG0071	Molecular chaperone (small heat shock protein)
26		-	24252	25778	ZP_08731588	DNA gyrase subunit B [Vibrio nigripulchritudo ATCC 27043]	0	94%	COG0187	Type IIA topoisomerase (DNA gyrase/topo II, topoisomerase IV) B subunit

B3TF_MPn_1 Annotation (31892 bp)

ORF	Gene	Strand	Start	End	Hit-Name	Description	E-Value	%-Ident	COG	Description
1		-	220	453	YP_581049	hypothetical protein Pcryo_1788 [Psychrobacter cryohalolentis K5]	2.00E-11	52%	-	-
2		+	704	1276	ZP_08330448	hypothetical protein IMCC1989_1237 [gamma proteobacterium IMCC1989]	2.00E-84	66%	COG1510	Predicted transcriptional regulators
3		+	1346	2728	ZP_01132291	quinol oxidase, subunit I [Pseudoalteromonas tunicata D2]	0	70%	COG1271	Cytochrome bd-type quinol oxidase, subunit 1
4		+	2738	3733	ZP_08330440	Cytochrome d ubiquinol oxidase subunit II [gamma proteobacterium IMCC1989]	1.00E-141	59%	COG1294	Cytochrome bd-type quinol oxidase, subunit 2

5		-	3727	4851	ZP_08330890	putative transport protein [gamma proteobacterium IMCC1989]	2.00E-96	44%	COG0477	Permeases of the major facilitator superfamily
6		-	4959	5609	ZP_08330885	Chloramphenicol acetyltransferase [gamma proteobacterium IMCC1989]	4.00E-76	71%	COG0110	Acetyltransferase (isoleucine patch superfamily)
7	*phnN*	-	5620	6228	ZP_08330884	ATP-binding protein PhnN [gamma proteobacterium IMCC1989]	3.00E-54	47%	COG3709	Uncharacterized component of phosphonate metabolism
8	*phnM*	-	6237	7379	ZP_01738680	hypothetical protein MELB17_10428 [Marinobacter sp. ELB17]	5.00E-156	57%	COG3454	Metal-dependent hydrolase involved in phosphonate metabolism
9	*phnL*	-	7376	8110	ZP_08330042	Phosphonates transport ATP-binding protein PhnL [gamma proteobacterium IMCC1989]	1.00E-107	66%	COG4778	ABC-type phosphonate transport system, ATPase component
10	*phnK*	-	8121	8912	ZP_08330041	Phosphonates transport ATP-binding protein PhnK [gamma proteobacterium IMCC1989]	6.00E-148	77%	COG4107	ABC-type phosphonate transport system, ATPase component
11	*phnJ*	-	8909	9775	ZP_08330037	PhnJ protein [gamma proteobacterium IMCC1989]	1.00E-173	86%	COG3627	Uncharacterized enzyme of phosphonate metabolism
12	*phnI*	-	9768	10961	ZP_08330036	PhnI protein [gamma proteobacterium IMCC1989]	0	63%	COG3626	Uncharacterized enzyme of phosphonate metabolism
13	*phnH*	-	10952	11572	ZP_08330035	PhnH protein [gamma proteobacterium IMCC1989]	6.00E-40	37%	COG3625	Uncharacterized enzyme of phosphonate metabolism
14	*phnG*	-	11590	12048	ZP_05067257	phosphonate C-P lyase system protein PhnG [Octadecabacter antarcticus 238]	7.00E-39	44%	COG3624	Uncharacterized enzyme of phosphonate metabolism
15	*phnE*	-	12177	12962	ZP_08330039	Phosphonate ABC transporter permease protein phnE [gamma proteobacterium IMCC1989]	7.00E-131	84%	COG3639	ABC-type phosphate/phosphonate transport system, permease component
16	*phnD*	-	13031	14020	ZP_08330040	Phosphonate ABC transporter phosphate-binding periplasmic component [gamma proteobacterium IMCC1989]	0	81%	COG3221	ABC-type phosphate/phosphonate transport system, periplasmic component
17	*phnC*	-	14041	14862	ZP_08330038	Phosphonate ABC transporter ATP-binding protein [gamma proteobacterium IMCC1989]	3.00E-147	70%	COG3638	ABC-type phosphate/phosphonate transport system, ATPase component
18		+	15051	15524	YP_001526312	GCN5-related N-acetyltransferase [Azorhizobium caulinodans ORS 571]	3.00E-44	45%	COG0454	Histone acetyltransferase HPA2 and related acetyltransferases
19	*phnF*	+	15534	15944	YP_957221	phosphonates metabolism transcriptional regulator PhnF [Marinobacter aquaeolei VT8]	8.00E-25	43%	COG2188	Transcriptional regulators
20	*phnP*	+	16119	16871	YP_004416487	carbon-phosphorus lyase complex accessory protein [Pusillimonas sp. T7-7]	2.00E-88	51%	COG1235	Metal-dependent hydrolases of the beta-lactamase superfamily I
21		-	16938	17894	YP_432296	hypothetical protein HCH_00984 [Hahella chejuensis KCTC 2396]	2.00E-109	52%	COG2307	Uncharacterized protein conserved in bacteria
22		-	17908	19365	YP_002513087	hypothetical protein Tgr7_1012 [Thioalkalivibrio sulfidophilus HL-EbGr7]	0	71%	COG2308	Uncharacterized conserved protein
23		-	19380	20261	ZP_08772441	transglutaminase domain protein [Thiocapsa marina 5811]	3.00E-108	53%	COG1305	Transglutaminase-like enzymes, putative cysteine proteases
24		-	20258	22885	ZP_08782045	protein of unknown function DUF404 [Methylobacter tundripaludum SV96]	0	45%	COG2308	Uncharacterized conserved protein
25		-	22950	26282	ZP_08782043	Protein of unknown function DUF2126 [Methylobacter tundripaludum SV96]	0	58%	COG4196	Uncharacterized protein conserved in bacteria
26		-	26689	28182	YP_002394921	ATP-dependent RNA helicase [Vibrio splendidus LGP32]	0	74%	COG0513	Superfamily II DNA and RNA helicases
27		+	28278	28694	YP_528670	hypothetical protein Sde_3201 [Saccharophagus degradans 2-40]	5.00E-64	72%	COG1186	Protein chain release factor B
28		-	28719	29285	YP_436149	hypothetical protein HCH_05041 [Hahella chejuensis KCTC 2396]	6.00E-79	65%	COG2840	Uncharacterized protein conserved in bacteria
29		-	29330	30262	YP_003809678	Transcriptional regulator, LysR family [gamma proteobacterium HdN1]	2.00E-112	52%	COG0583	Transcriptional regulator
30		+	30479	31864	ZP_01075298	hypothetical protein MED121_14060 [Marinomonas sp. MED121]	1.00E-148	51%	COG1033	Predicted exporters of the RND superfamily
